# Highly Selective Polyelectrolyte Multilayer Membranes Through Hydrophobic Interactions

**DOI:** 10.1021/acsami.4c20150

**Published:** 2025-03-27

**Authors:** Wendy A. Jonkers, Maxime Precheur, J. Roberto Andrade, Wiebe M. de Vos, Esra te Brinke

**Affiliations:** Membrane Science and Technology, University of Twente, MESA+ Institute for Nanotechnology, P.O. Box 217, 7500 AE Enschede, The Netherlands

**Keywords:** membrane, nanofiltration, hydrophobicity, polyelectrolyte multilayer, wastewater treatment, organic micropollutants

## Abstract

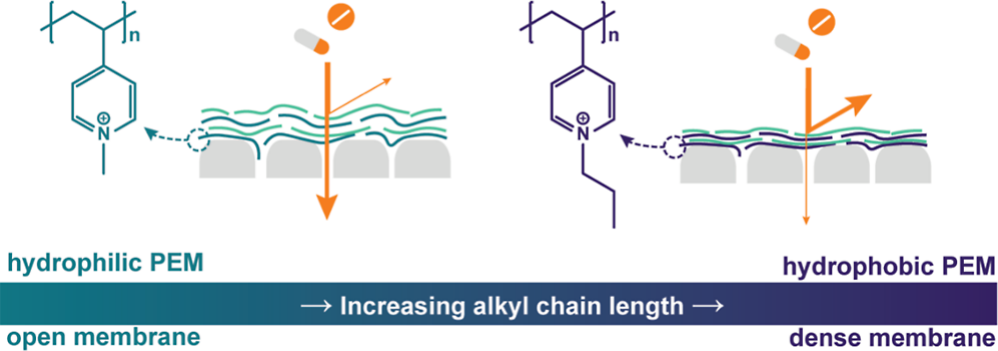

Polyelectrolyte multilayer (PEM) membranes are highly promising for the removal of organic micropollutants (OMPs) from wastewater. However, for the removal of small OMPs, dense membranes with a low molecular weight cutoff (MWCO) are required. It has been recently demonstrated that MWCO correlates with PEM swelling by water. We therefore propose that dense membranes could be fabricated by enhancing hydrophobic interactions to decrease swelling. Controlled synthesis of hydrophobic polycations was achieved by quaternization of poly(4-vinylpiridine) (P4VP) with alkyl chains of varying length, which will also enhance its chemical stability. Optical reflectometry shows that multilayers can be successfully grown with the quaternized P4VPs (QP4VPs) and poly(styrenesulfonate) (PSS). Permeability and MWCO tests demonstrate that the membrane density can indeed be increased by increasing the length of the alkyl chain. Polycation terminated membranes are denser than polyanion membranes, likely due to the higher ratio of hydrophobic polycation. Propyl-QP4VP/PSS membranes have a MWCO as low as 230 Da. In line with the MWCO, OMP retention increases with increasing alkyl chain length. The QP4VP membranes studied here outperform commonly used poly(diallyldimethylammonium chloride) PDADMAC/PSS membranes in selectivity and are made with more chemically stable polyelectrolytes than other dense PEM membranes. The presented research confirms that PEM density correlates with hydrophobicity, and that PEM membranes can be densified through the addition of alkyl chains.

## 1 Introduction

Since their inception in the 1990s,^1^ polyelectrolyte multilayers (PEMs) have proven to be versatile materials with a vast range of applications.^2−5^ One area where PEMs have shown remarkable success is membrane technology. Here, support membranes coated with PEMs have surpassed the laboratory stage, and are already practically applied in commercial settings.^6^ Currently, the densest commercially available PEM membranes have a molecular weight cutoff (MWCO) of 400 Da,^6^ but there is a particular interest within the scientific community to develop denser PEM membranes with a lower MWCO. Dense PEM membranes can for example be employed in the removal of organic micropollutants from wastewater.^7,8^

A frequently used approach to create denser PEM membranes is to increase the extent of electrostatic interactions of the PEM. This can be done by increasing the amount of intrinsic charge compensation by coating at low salt concentrations^8,9^ or by optimizing the pH in the case of pH-sensitive polyelectrolytes.^10−12^ Another strategy is to use polyelectrolytes with a high charge density, either by using a different chemistry,^3,13^ or by increasing the ratio of charged monomer units.^14^ However, a recent experimental review that compared nine polyelectrolyte pairs highlighted that the charge density of the used polyelectrolyte pair and the MWCO of the PEM membrane are not correlated,^15^ showing that creating PEM membranes with a low MWCO is more complicated than initially assumed. This is likely due to the contribution of other intermolecular interactions, besides ionic cross-linking, which play a role in PEM buildup.

Indeed, while electrostatic interactions are pivotal for the buildup for a polyelectrolyte multilayer, it has been long understood that there are many more types of interactions which can play a role during layer-by-layer assembly, though not all these interactions are well studied in the context of membranes. Other molecular interactions which can play a role are hydrogen bonding, hydrophobic interactions, charge transfer, host–guest coordination chemistry (i.e., π–π and π–cation interactions), and covalent bonding.^16^ While many of these interactions are weaker in magnitude than electrostatic interactions, it is important to remember that this effect is additive to the electrostatic interactions present in PEM layers and can thus strengthen or weaken the overall interaction. As an illustration, PEM layers with a polycation charge density as low as 8 mol % can be grown because of nonelectrostatic interactions.^17^ More so, PEM membranes have even been fabricated with uncharged polymers, through hydrogen bridges and hydrophobic interactions, albeit with a very low permeability.^18^ Especially these hydrophobic interactions can have a substantial contribution to the overall binding energy.^19^

We propose optimizing hydrophobic interactions may prove advantageous for creating denser PEM membranes. The main driver for hydrophobic interactions is stated to be entropic gain upon release of water molecules when hydrophobic polyelectrolytes interact,^19,20^ though enthalpy may play a role as well.^21^ Indeed, it has been shown that more hydrophobic multilayers contain less water,^20^ and that hydrophobic polyelectrolyte complexes are less prone to swelling.^22^ This can be advantageous for creating denser membranes, as membrane swelling has shown to be a better predictor for MWCO than the charge density of the used polyelectrolyte pair.^15^ In order to increase the strength of the hydrophobic interactions, hydrophobic side groups can be added to the polyelectrolytes, for example by attaching alkyl chains of increasing chain length to polyelectrolytes.^22,23^

However, a dense separation layer is not the only requirement for PEM membranes. A very important requirement for commercial use is chemical stability of the used polyelectrolytes. In industrial applications, membranes are frequently cleaned with hypochlorite,^24,25^ which is used for the oxidation of organic foulants. Unfortunately, hypochlorite can damage the PEM layer over time.^26^ In polycations, the positive charge is frequently introduced through an amine group. It has been demonstrated that quaternary amines are much more resistant against oxidation by hypochlorite than primary, secondary and tertiary amines, as the quaternary amines are already in their lowest redox state.^26,27^ In addition, quaternary amines are strong (permanently charged) polyelectrolytes. The use of strong polyelectrolytes also guarantees a higher pH stability than the use of pH-sensitive, weak polyelectrolytes.^28^ Thus, the use of quaternary amines is advantageous as it yields higher stability to both hypochlorite and extreme pH conditions. An example of such a system with a high stability to both chemicals and hypochlorite is poly(diallyldimethylammonium chloride) (PDADMAC)/poly(4-styrenesulfonate) (PSS).^26,28^ However, this system does not yield very dense membranes.^15^ We propose that the requirement for both dense and stable membranes could be met by using a polyelectrolyte with both a quaternary amine group and a hydrophobic alkyl chain and hypothesize that PEM density will increase with alkyl chain length.

In this work, strong hydrophobic polycations will be synthesized by the quaternization of poly(4-vinylpyridine) (P4VP).^22^ Alkyl chains of different lengths will be used for quaternization to induce different extents of hydrophobicity. The hydrophobicity of the multilayers is determined with contact angle measurements. Multilayers of P4VP or quaternized P4VP (QP4VP) and the commonly used polyanion PSS will be built. Though limited research has been done regarding P4VP/PSS^3,29,30^ and methyl-QP4VP/PSS,^31,32^ their use in nanofiltration membranes has been mostly unexplored. The well studied PDADMAC/PSS is used here as a reference system. Growth of the multilayers on model surfaces is studied with optical reflectometry. Then, PEM membranes are fabricated by dip-coating the same systems onto hollow fiber support membranes. Subsequently, permeability, MWCO, salt and OMP retention are determined on a crossflow setup. Overall, we will clearly demonstrate that dense PEM membranes can be fabricated through increasing the hydrophobic interactions in the PEM layer.

## 2 Materials and Methods

### 2.1 Chemicals

NaCl was obtained from Nobian (The Netherlands), 2-component epoxy glue (2K Expert) was obtained from Bison. Iodoethane, LC/MS grade methanol and ethanol (99%) were obtained from Fisher Scientific. Poly(diallyldimethylammonium chloride) (PDADMAC, 200–250 kDa, 20 wt % in water), iodomethane, 1-iodopropane, Na_2_SO_4_, deuterium oxide, deuterium chloride, sodium hypochlorite (6–14% active chlorine), ethylene glycol and polyethylene glycol (PEG) 200, 400, 600, 1000, 1500, and 2000 were obtained from Merck. Hollow fiber support membranes (inner diameter = 0.7 mm, MWCO = 10,000 Da) were kindly donated by NX Filtration (The Netherlands). Acetic acid was obtained from Boom B.V.; Milli-Q water was produced in-house. Poly(4-vinylpyridine) (P4VP, 200 kDa) was obtained from Scipoly(USA). MgCl_2_, MgSO_4_, poly(styrenesulfonate (PSS, 200 kDa, 30 wt % in water), glycerol (83.5–89.5%), dialysis membrane (cellulose, MWCO = 14,000 Da), ammonium acetate, diethylene glycol, triethylene glycol, salicylic acid, ibuprofen, naproxen, sulfamethoxazole, diclofenac, bezafibrate, pyrazole, benzotriazole, caffeine, isoproturon, atrazine, bisphenol A, carbamazepine, phenolphthalein, bromothymol blue, metformin, lidocaine, atenolol, metoprolol tartrate and nadolol were obtained from Sigma-Aldrich. Paracetamol, sotalol and amisulpride were obtained from British Pharmacopoeia (United Kingdom). Dimethyl sulfoxide (DMSO, 99%) was obtained from Thermo Scientific.

### 2.2 Synthesis

**Figure 1:**
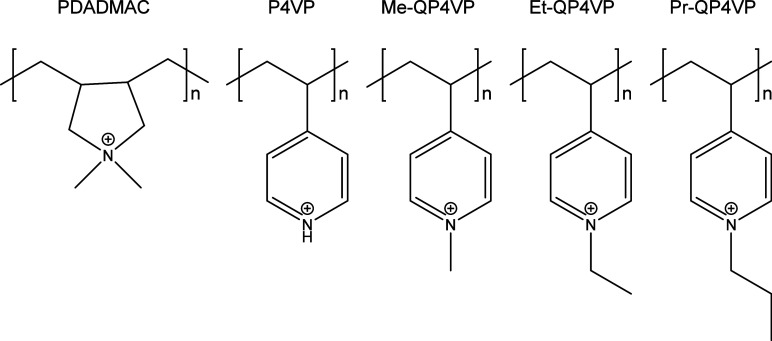
Structures of the polycations used in this study.

In this work, we refer to methyl-, ethyl- and propyl-substituted P4VP as Me-QP4VP, Et-QP4VP and Pr-QP4VP, respectively (Figure 1). For the synthesis of the fully substituted QP4VPs, a previously published protocol was adapted.^22^ 4.00 g of P4VP was dissolved in 200 mL DMSO. Then, the mixture was heated, and a 500% molar excess of an alkyl halide was added. The reaction temperature and added alkyl halide differed for each synthesis: iodomethane (room temperature) for Me-QP4VP, iodoethane (50 °C) for Et-QP4VP and 1-iodopropane (80 °C) for Pr-QP4VP. The solution was stirred for 24 h. After the reaction was completed, the reaction mixture was purged with N_2_ for 3 h to remove the excess alkyl halide. Then, the reaction mixture was precipitated in 300 mL ethanol, and the solvent was removed by vacuum filtration. The precipitate was dissolved in 1.0 M NaCl in water.

Dialysis was performed to replace the iodide counterions with chloride counterions and to remove ions not participating in extrinsic compensation. First, dialysis was performed for 1.5 days in 5 L 0.2 M NaCl, followed by 4 days in 5 L Milli-Q water. The external solution was replaced twice daily. Then, dry polymer was obtained by evaporating the solvent in an oven at 110 °C. An ^1^H NMR spectrum of P4VP in deuterium oxide with deuterium chloride was measured on a Bruker Ultrashield 600 MHz NMR spectrometer. ^1^H NMR spectra of the substituted compounds in deuterium oxide (residual solvent peak at δ 4.79) were measured on a Bruker Ascend 400 MHz NMR spectrometer. The spectra are displayed in the Supporting Information, Figure S1.

**P4VP**: ^1^H NMR (D_2_O with DCl, 600 MHz) δ 1.39 (br, 3H), 2.13 (br, 0.2H), 6.84 (br, 2H), 7.84 (br, 2H). **Me-QP4VP**: ^1^H NMR (D_2_O, 400 MHz) δ 1.97 (br, 3H), 4.25 (br, 3H), 7.50 (br, 2H), 8.49 (br, 2H). **Et-QP4VP**: ^1^H NMR (D_2_O, 400 MHz) δ 1.53 (br, 3H), 2.04 (br, 3H), 4.48 (br, 2H), 7.45 (br, 2H), 8.57 (br, 2H). **Pr-QP4VP**: ^1^H NMR (D_2_O, 400 MHz) δ 0.70 (br, 3H), 1.25–2.45 (br, 2H + 3H), 2.80 (br, 2H), 4.44 (br, 2H), 7.61 (br, 2H), 8.62 (br, 2H).

### 2.3 Contact Angle

The hydrophobicity of the PEMs was studied with contact angle measurements. Silicon wafers with an 81 nm silicon dioxide surface layer were used as a model substrate. To remove any contamination of the surface, the wafers were pretreated by flushing them three times with water and ethanol, followed by a plasma treatment with O_2_ plasma for 15 min in a diener electronic Femto plasma cleaner. Then, the PEM coating was applied to the wafers. For the coating, polyelectrolyte solutions were prepared containing 0.1 g/L polyelectrolyte and 50 mM NaCl in Milli-Q water. The pH of the P4VP solution was adjusted to 1.5 in order to dissolve P4VP. The washing solution consisted of 50 mM NaCl in Milli-Q water. The wafers were mounted in a holder and then the holder was immersed in polycation solution for 15 min. Then, they were submerged three times 5 min in a washing solution. Next, they were immersed in polyanion solution for 15 min, followed by three washing steps. This marks the formation of the first bilayer on the wafers. The process was repeated until 2.5 bilayers had been deposited.

The contact angle measurements were performed on a on a Dataphysics Contact angle system OCA using the sessile drop method, and the contact angle was determined using SCA20 software. For each wafer, three droplets of 2.0 μL were deposited as a technical replicate. Three wafers were measured per coating type.

### 2.4 Reflectometry

The growth of the PEMs on model surfaces was studied with fixed angle optical reflectometry. For each PEM layer, the experiment was repeated three times. The theoretical background for reflectometry measurements has been extensively described elsewhere.^33^ Silicon wafers with an 81 nm silicon dioxide surface layer were used as a substrate. They were pretreated in the same manner as the wafers for the contact angle measurements. Then, the wafers were mounted in a stagnation point flow cell. During the measurement, polycation, washing and polyanion solutions are alternately introduced to the flow cell. The coating solutions all contained 50 mM of NaCl, and 0.1 g/L polyelectrolyte. For P4VP, the pH was set to 1.5 in order to dissolve the P4VP. PSS, Me-QP4VP, Et-QP4VP and Pr-QP4VP are strongly charged polyelectrolytes, and for these polyelectrolytes the pH was left unadjusted. Washing solution consisted of 50 mM NaCl in water. A switch to a new solution is made when the signal has been stabilized.

Adsorption is monitored by measuring the ratio of the p- and s-polarized components of the light of a laser beam (632.8 nm) which is reflected onto the surface. From the change in this ratio (Δ*S*), the amount of adsorbed mass on the wafer can be calculated with formula 1
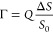
1Here, Γ is the mass of the adsorbed polyelectrolyte in mg m^–2^, *Q* is the sensitivity factor of the polyelectrolyte in mg m^–2^, Δ*S* is the change in the ratio of the polarized light components in mV, and *S*_0_ is the starting value of the ratio of the polarized light in mV.

The sensitivity factor Q of each polyelectrolyte was calculated with an optical model, which used as input parameters the wavelength (632.8 nm) and angle of incidence of the later beam (71°), the thicknesses of the silicon dioxide layer (81 nm) and the polyelectrolyte multilayer (The thickness of the polyelectrolyte multilayer increases during the experiment. It is assumed to be 20 nm for the purpose of the model), the refractive indices of silicon (3.85), silicon dioxide (1.46) and the solvent (1.33) and the d*n*/d*c* of the used polymers. The d*n*/d*c* values for P4VP (0.2933 mL g^–1^), Me-QP4VP (0.2053 mL g^–1^), Et-QP4VP (0.1851 mL g^–1^) and Pr-QP4VP (0.2142 mL g^–1^) were determined with a SCHMIDT HAENSCH ATR-lambda refractometer. A detailed description of this determination can be found in the Supporting Information, Figure S2. This resulted in *Q* factors for P4VP (16.9 mg m^–2^), Me-QP4VP (24.1 mg m^–2^), Et-QP4VP (23.1 mg m^–2^) and Pr-QP4VP (25.2 mg m^–2^). The *Q* factor for PSS in these conditions was previously determined to be 30.1 mg m^–2^.^34^

### 2.5 Membrane Coating

In order to assess the effect of the hydrophobic coatings on membrane performance, hollow fiber ultrafiltration support membranes were coated with the layer-by-layer technique. The support membranes are asymmetric, having denser pores on the inside of the fiber than on the outside of the fiber. Field-emission scanning electron microscopy (FE-SEM) images of the support membranes are displayed in Supporting Information, Figure S3. As preparation, the support membranes were cut to approximately 32 cm length, and were immersed overnight in 10% ethanol in water to remove glycerol left from the production process. Afterward, the fibers were immersed in Milli-Q water. The coating procedure for the membranes was similar to the coating procedure used for the wafers for the contact angle measurements. However, the membranes were coated with a dipcoat robot, and the number of bilayers that was coated varied for each membrane and was derived from the reflectometry analysis, aiming for 10 mg m^–2^ of deposited PEM material.

After coating, the membranes are deposited in Milli-Q water for 15 min and subsequently into an aqueous solution containing 15 wt % glycerol for a minumum of 4 h to prevent pore collapse. The membranes are dried overnight in a controlled airflow. Finally, the membranes were potted into single-fiber modules. For this, the membranes were placed into 24 cm long plastic tubing, with a hole cut at the midpoint for permeate collection. The membranes were glued into place with epoxy glue, and dried overnight. Before testing, the ends of the module were cut off to achieve a clean edge, and the active length of the membrane was measured with a ruler. Four modules were produced per PEM coating type.

### 2.6 Membrane Performance Measurements

Permeability, molecular weight cutoff (MWCO), salt retention and OMP retention of the coated membranes were measured on a custom-build crossflow setup. In the crossflow setup, 16 fibers could be measured in parallel, with four modules measured per type of PEM coating. Before the first measurement, membranes were flushed with Milli-Q water for 30 min to remove the glycerol. After each measurement, the membranes were flushed for 20 min with Milli-Q water to clean them. During all measurements, the feed temperature was set to 20 ± 1 °C, The crossflow velocity was set to 1 m s^–1^, the pressure was set to 1.9 bar. Four modules were measured per coating as replicates for all experiments. A Gaussian distribution was assumed for the calculation of the mean values and 95% confidence intervals.

#### 2.6.1 Pure Water Permeability

For the pure water permeability (PWP) test, Milli-Q water was used as a feed. The experiment was ran for 1 h, and permeate was collected in tubes. Those tubes were measured before and after running the test, to determine the mass of the collected permeate. The permeability was then determined using formula 2
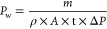
2Here, *P*_w_ is the permeability in L m^–2^ h^–1^ bar^–1^, m is the mass of the permeate in g, ρ is the density of pure water (assumed to be 1.00 g L^–1^), *A* is the surface area of the membrane in m^2^, *t* is the duration of the permeation in h, and Δ*P* is the transmembrane pressure in bar.

#### 2.6.2 Molecular Weight Cut-off

For the molecular weight cut-off (MWCO) experiment, a feed mixture was prepared containing ethylene glycol, diethylene glycol, triethylene glycol and polyethylene glycol with a molecular weight of 200, 400, 600, 1000, 1500 and 2000, each at a concentration of 1 g L^–1^. Permeate was collected for 30 min, and a feed sample was collected 15 min after the start of permeate collection. The collected samples were analyzed with gel permeation chromatography (GPC), on an Agilent 1200/1260 Infinity GPC/SEC series apparatus containing two columns in series (Standers Service Suprema 8 × 300 mm: 1000 Å, 10 μm, followed by 30 Å, 10 μm). The retention was calculated using formula 3
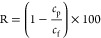
3Here, *R* is the retention in %, *c*_p_ is the concentration of the compound in the permeate, and *c*_f_ is the concentration of the compound in the feed. The 90% MWCO was determined by interpolation of the retention data.

#### 2.6.3 Salt Retention

For the salt retention measurements, single-salt solutions (NaCl, Na_2_SO_4_, MgCl_2_ or MgSO_4_) at a concentration of 5 mM were prepared. Permeate was collected over the course of 2.5 h. The conductivity of the feed was measured before and after the measurements with a Xylem Analytics WTW Profline Cond3310 conductivity meter. The conductivity of the permeate was measured at the end of the experiment. The salt retention was then calculated using formula 4
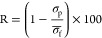
4Here, *R* is the retention in %, σ_p_ the conductivity of the permeate and  the average conductivity of the feed in μS cm^–1^.

#### 2.6.4 Organic Micropollutant Retention

For the organic micropollutant (OMP) measurements, an OMP feed mixture was prepared containing the OMPs salicylic acid, ibuprofen, naproxen, sulfamethoxazole, diclofenac, bezafibrate, pyrazole, benzotriazole, paracetamol, caffeine, isoproturon, atrazine, bisphenol A, carbamazepine, phenolphthalein, bromothymol blue, metformin, lidocaine, atenolol, metoprolol, sotalol, nadolol and amisulpride, at a concentration of 50 μg L^–1^ (salicylic acid and bromothymol blue at 250 μg L^–1^ because of significant degradation over the course of the experiment) in Milli-Q water with 5 mM NaCl, pH 5.8. To account for OMP adsorption to the membrane and filtration system, the OMP mixture was first filtrated for 28 h. During this time, concentrate and permeate was recirculated to the feed solution. After this equilibration stage, feed and permeates were collected for 15 min.

Sample analysis was performed using a high-performance liquid chromatography–mass spectrometry (HPLC–MS) system. The HPLC system (Thermo Scientific, UltiMate 3000 RSLC) was equipped with an Acclaim RSLC Polar Advantage column (Thermo Scientific, 100 × 2.1 mm, 2.2 μm particle size) with AccuCore C18 guard (Thermo Scientific, 10 × 2.1 mm, 2.6 μm particle size). OMPs were separated over a gradient with 1.85 mM ammonium acetate and 0.15 mM acetic acid in Milli-Q water versus LC/MS grade methanol at a flow rate of 0.5 mL/min. Mass spectrometry was performed with a triple quadrupole mass spectrometer (Thermo Scientific, TSQ Quantis), in positive (5 μL injection volumes) and negative (50 μL injection volumes) ionization mode, using heated electrospray ionization (pyrazole and metformin: 2500 V, 400 °C; other compounds in positive ionization mode: 4000 V, 400 °C; negative ionization mode: 3500 V, 275 °C) and in single reaction monitoring (MS/MS) mode.

OMP retention was quantified on the most intense precursor–product transition of each OMP with Chromeleon 7 software, using a calibration curve. The calibration curve was created by diluting a feed sample (1, 2, 10, 20, 100, 200, and 1000 times diluted), which was obtained during permeate collection. To account for possible sensitivity differences during the HPLC–MS analysis, the calibration series was analyzed both before and after the permeate samples in each ionization mode. The peak areas of the permeate and calibration samples were integrated, and retentions were calculated from the average of the two calibration curves.

## 3 Results and Discussion

### 3.1 Synthesis

Me-QP4VP, Et-QP4VP and Pr-QP4Vp were successfully synthesized by a reaction with an iodo-alkane (iodomethane, iodoethane and 1-iodopropane, respectively) (Supporting Information, Figure S1). Full (100%) quaternization could be achieved for all these compounds when the reaction was performed in the solvent DMSO. Partial (75%) quaternization can be achieved for Me-QP4VP when the reaction is performed in water. This work focuses on the use of fully quaternized P4VP, for more information on partially quaternized Me-QP4VP, we refer to the Supporting Information.

### 3.2 Multilayer Growth

**Figure 2:**
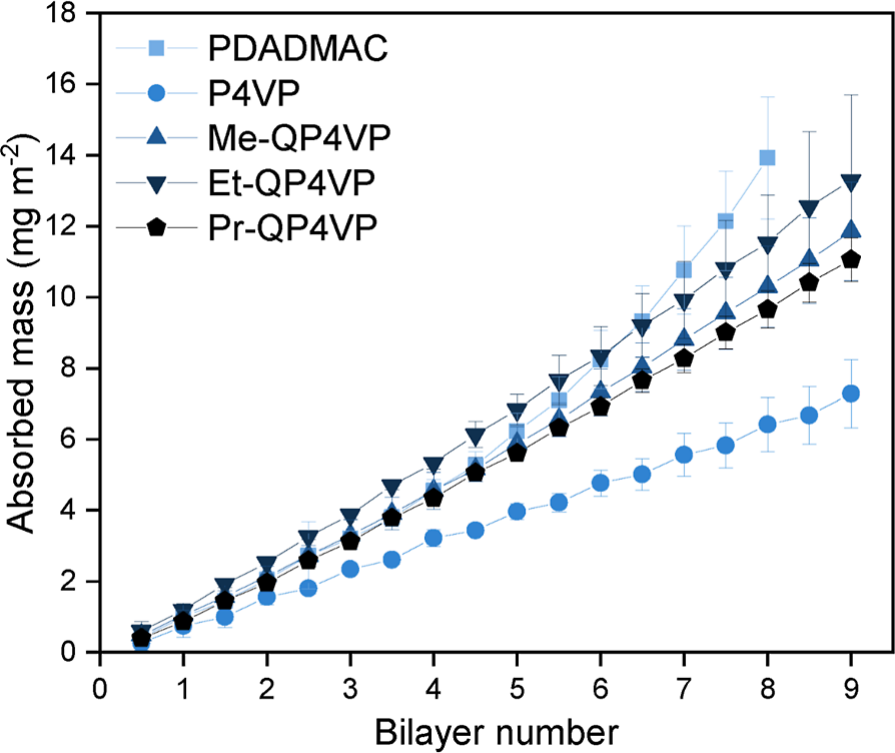
Layer growth of polyelectrolyte multilayers studied with reflectometry. Error bars represent the 95% confidence interval (*n* = 3). PDADMAC data was previously published in literature.^15^

To get an impression of the number of bilayers required for membrane coating, multilayers were coated on model surfaces and their growth was studied with optical fixed angle reflectometry (Figure 2). Silicon wafers were used as model surfaces, P4VP and its quaternized variants were used as polycations, and PSS was used as a polyanion. Data for PDADMAC/PSS layer growth was derived from a previously published paper,^15^ where layer growth was studied under the same conditions (0.1 g L^–1^ polyelectrolyte, 50 mM NaCl).

**Figure 3:**
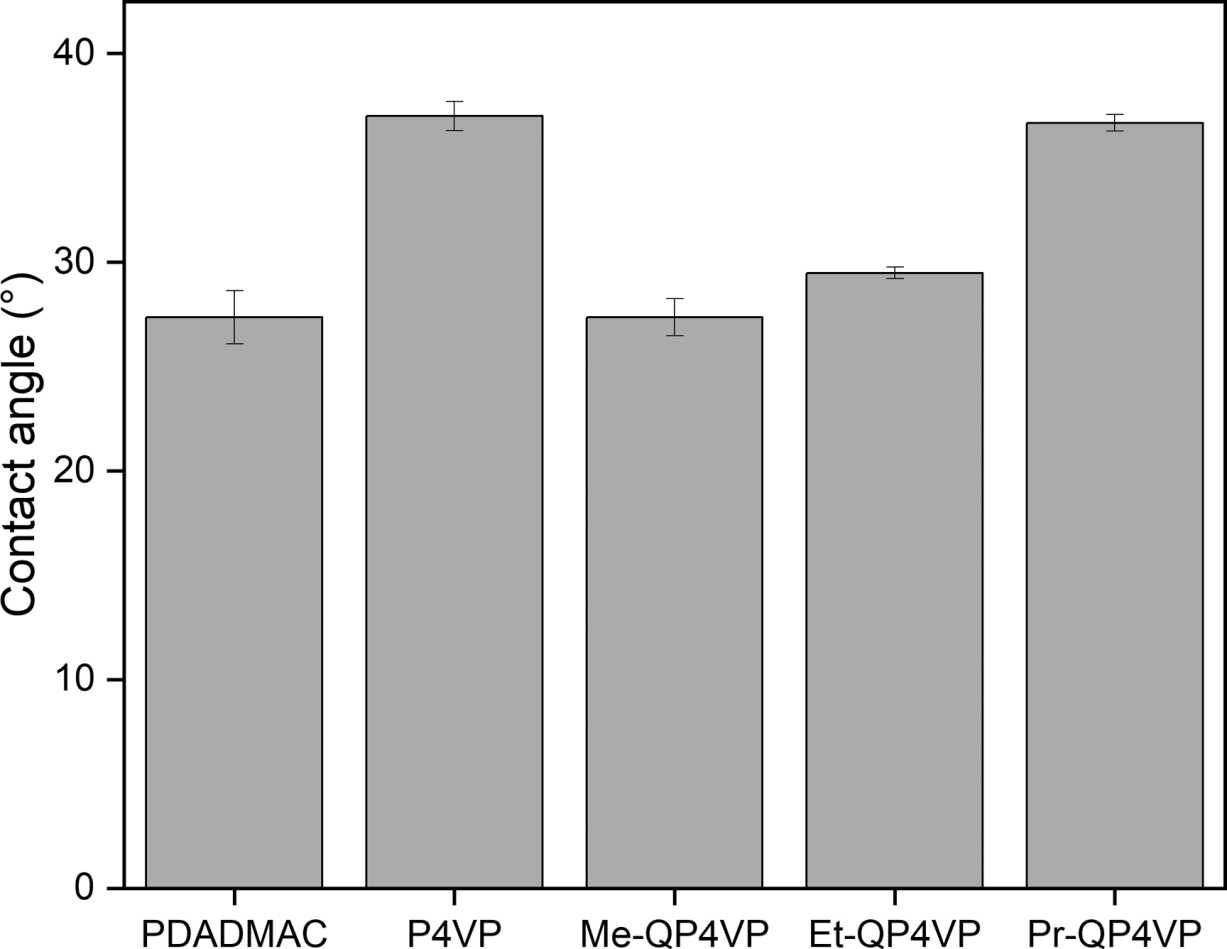
Contact angle of polyelectrolyte multilayers on model substrates as determined with sessile drop. All multilayers consist of 2.5 bilayers and contain poly(styrenesulfonate) as a polyanion. Error bars represent the 95% confidence interval (*n* = 3).

The reflectometry data demonstrate that multilayers can successfully be constructed with the QP4VPs (Figure 2). All PEMs constructed with QP4VPs show linear growth, indicating a low mobility of the polyelectrolytes within the multilayer.^16^ The growth rates of PEMs constructed with QP4VPs were nearly identical, with differences in adsorption primarily falling within the range of experimental error. Previous literature suggests that longer alkyl chain lengths correlate with increased growth rates due to the formation of hydrophobic domains.^37^ This effect was not observed within this study, potentially because of the use of different polymers.

The growth curves help in determining how many bilayers of the PEM should be applied in membrane coating. When there are only a few bilayers, the pores of the support membrane are not fully sealed, and the membrane properties are mainly influenced by the resulting pores (pore-dominated regime).^9^ To use the PEM as a separation layer that determines the membrane properties, we need to apply enough bilayers to seal the pores of the membrane (entering the layer-dominated regime). The number of layers required to enter the layer-dominated regime depends strongly on the used polyelectrolyte pair and support membrane.^15^ However, for the support membranes used for this study, the layer-dominated regime is typically achieved when the adsorption exceeds 10 mg m^–2^.^15^ For a fair comparison among different membranes, it is best to aim for an equal amount of adsorbed mass in each multilayer. The similar growth rate meant that an equal number of bilayers could be used to coat the QP4VPs. In order to ensure proper transition to the layer-dominated regime, 9 (negatively terminated) and 9.5 (positively terminated) bilayers were sufficient for these polycations.

The multilayer consisting of P4VP/PSS also shows linear growth, albeit with a much lower growth rate than for the QP4VPs, likely due to a low mobility of P4VP. Here it is important to mention that P4VP was coated at a pH of 1.5, as at that pH it is positively charged and water-soluble, while at higher pH it is uncharged and water insoluble. To get an estimate of the required number of bilayers for this membrane, the growth curve was extrapolated. It was determined that for an equal mass adsorption as for the QP4VPs, 16 bilayers should be coated for the P4VP/PSS system. Therefore, 16 (negatively terminated) and 16.5 (positively terminated) bilayers were coated for this system.

As previously reported in literature,^9,15^ multilayers can also be successfully constructed with the PDADMAC reference system. The PEM initially grows linearly, but a slight deviation from linearity can be observed at higher layer numbers (4.5 bilayers onward), indicating an extent of interpenetration for these layers. Defect-free membranes have been produced for PDADMAC/PSS membranes with 8 bilayers,^38^ hence 8 and 8.5 bilayers were coated for this system.

### 3.3 Contact Angle

To assess the hydrophobicity of the PEMs, the contact angle of polycation-terminated multilayers was measured with a sessile drop method (Figure 3). A clear trend is visible for the QP4VPs, with Me-QP4VP having the lowest contact angle (27.4 ± 0.9°), followed by Et-QP4VP (29.5 ± 0.3°), and Pr-QP4VP having the highest contact angle (36.7 ± 0.1°). In the sessile drop method, a higher contact angle indicates a more hydrophobic material. This demonstrates that a longer alkyl chain in the polycation leads to a more hydrophobic multilayer for polycation-terminated multilayers. A similar trend has been reported in the literature for QP4VP/carboxymethylcellulose PEMs,^35^ indicating that this behavior is observed with other polyanions as well. In our experiment, only polycation-terminated PEMs were measured. It is known that the contact angle of a PEM can vary depending on the terminating polyelectrolyte, with polyanion-terminated layers generally being more hydrophilic.^36^

P4VP does not follow the same trend as the QP4VPs. Based on its molecular structure, it could be expected that P4VP would be more hydrophilic and have a lower contact angle than Me-QP4VP, as it lacks the hydrophobic alkyl chain. However, the contact angle of P4VP is much higher (37.0 ± 0.7°), and is more similar to Pr-QP4VP. This shows that P4VP-terminated multilayers are relatively hydrophobic. It should be noted that for the P4VP-terminated surfaces, the contact angle was decreasing during the measurement due to spreading of the droplets over the surface. This complicated accurate determination of the contact angle. The deviation of the P4VP membranes from the general trend could be explained by the very complex nature of the P4VP/PSS system. The P4VP/PSS wafers were coated at pH = 1.5. This is to ensure that the pH-sensitive P4VP is sufficiently charged to form multilayers. However, contact angle tests are executed with Milli-Q water, which has a pH around 5.5–6. It is likely that P4VP partially loses its charge at this pH and thereby becomes more hydrophobic. Contrastingly, the contact angle for the PDADMAC multilayers (27.4 ± 1.3°) is similar to the contact angle of the Me-QP4VP multilayers, showing that PDADMAC/PSS multilayers are comparatively hydrophilic.

### 3.4 Permeability and MWCO

Subsequently, membranes were coated with multilayers of PDADMAC/PSS, P4VP/PSS or QP4VP/PSS, with the number of deposited multilayers as determined by the reflectometry experiments. Field-emission scanning electron microscopy (FE-SEM) images reveal the deposition of a multilayer across all systems, but no substantial differences in structure were observed between the membranes (Supporting Information, Figure S3). However, this lack of visible variation may be attributed to the resolution limitations of the SEM, given the small height of the multilayer. The permeability and MWCO of the membranes were tested in a crossflow setup.

**Figure 4:**
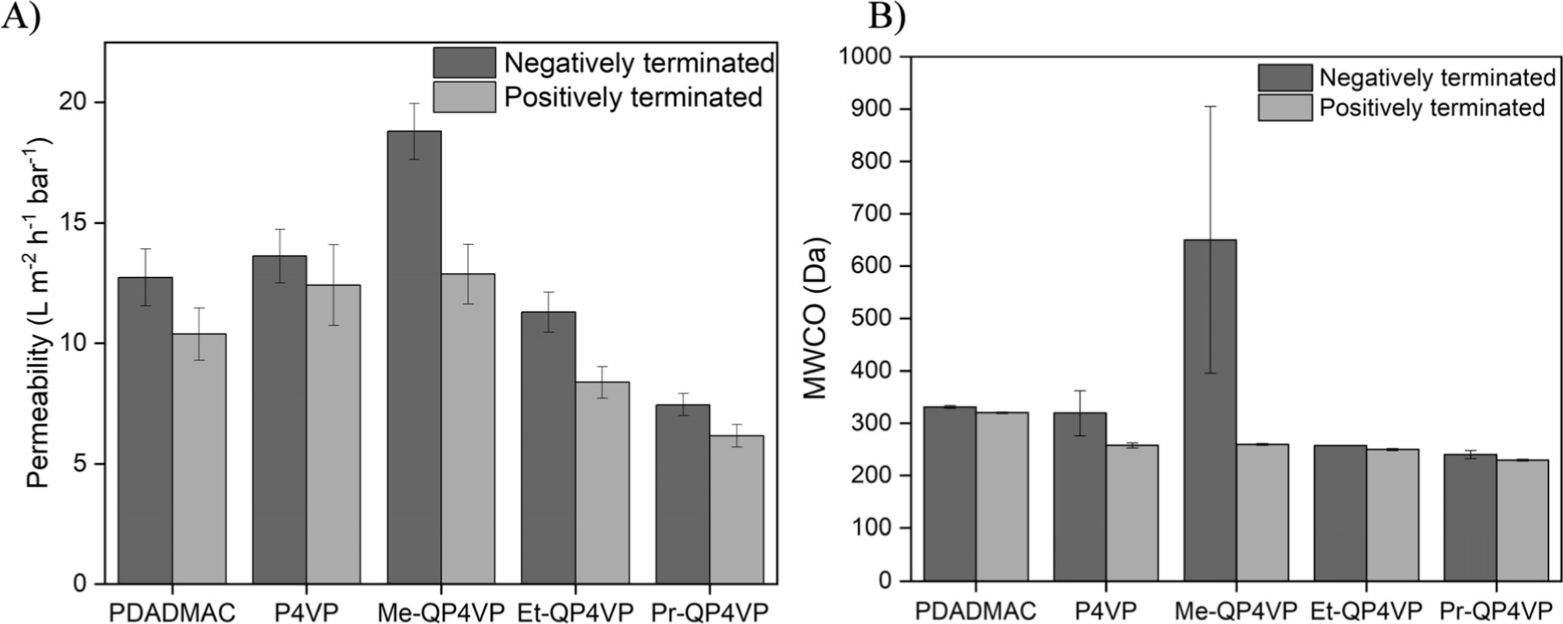
(A) Permeability of PEM membranes used in this study. (B) 90% MWCO of PEM membranes used in this study. PDADMAC data was previously published in ref (15). Error bars represent the 95% confidence interval (*n* = 4).

A clear trend in permeability is visible for both the negatively and the positively terminated QP4VP membranes (Figure 4A). The permeability of QP4VP membranes decreases as the alkyl chain length increases. This trend can be attributed to the enhanced hydrophobicity of the polycation as the alkyl chain length increases, likely leading to less swollen membranes.

Another visible trend is that the positively terminated QP4VP membranes consistently show a lower permeability than the negatively terminated membranes. Especially for Me-QP4VP, the permeability decreases from 18.8 ± 1.2 L m^–2^ h^–1^ bar^–1^ for [Me-QP4VP/PSS]_9_ to 12.9 ± 1.3 L m^–2^ h^–1^ bar^–1^ for [Me-QP4VP/PSS]_9.5_, a substantial decrease. The positively terminated membranes do contain half a bilayer of additional coating, which increases the thickness of the separation layer and can therefore contribute to the hydraulic resistance. However, the increased thickness of the separation layer cannot explain the big differences in permeability which are observed between the negatively and positively terminated membranes, especially since the growth of the multilayer is linear, as demonstrated with reflectometry. A more likely cause is that the positively terminated membranes contain a higher ratio of the hydrophobic QP4VPs and thus swell less. Such odd–even effects are more commonly observed in PEM membranes.^9^

The negatively terminated PDADMAC (12.7 ± 1.2 L m^–2^ h^–1^ bar^–1^) and P4VP (13.6 ± 1.1 L m^–2^ h^–1^ bar^–1^) membranes have a lower permeability than the negatively terminated Me-QP4VP membrane (18.8 ± 1.2 L m^–2^ h^–1^ bar^–1^). The lower permeability of the PDADMAC system could be a consequence of a thicker separation layer. As could be seen in Figure 2, the PDADMAC/PSS system has a higher mass adsorption than the QP4VP/PSS systems at high layer numbers. This can lead to a thicker separation layer and thereby an increased hydraulic resistance. Meanwhile, the positively terminated PDADMAC (10.4 ± 1.1 L m^–2^ h^–1^ bar^–1^) and P4VP (12.4 ± 1.7 L m^–2^ h^–1^ bar^–1^) membranes have a comparable permeability to the positively terminated Me-QP4VP membrane (12.9 ± 1.3 L m^–2^ h^–1^ bar^–1^).

Looking at the MWCO for the negatively terminated membranes (Figure 4B), a dependency of the MWCO on alkyl chain length can be observed. For Me-QP4VP, a rather high MWCO of 650 ± 254 Da is observed, which decreases to 257 ± 1 Da for Et-QP4VP and 240 ± 8 Da for Pr-QP4VP. The rather large error bars for the negatively terminated P4VP and especially Me-QP4VP stand out. These membranes were studied in more detail by looking at the sieving curves for the MWCO measurements (Supporting Information, Figure S4). The sieving curves indicate that some defects are present in the negatively terminated P4VP and Me-QP4VP membranes and show that these membranes are not very reproducible in terms of defects. The defects might also explain the rather high permeability for the negatively terminated Me-QP4VP membrane. The negatively terminated Et-QP4VP and Pr-QP4VP membranes appear to be defect-free. The defects in the negatively terminated Me-QP4VP membranes can be prevented by coating another bilayer of Me-QP4VP/PSS on top of the membrane (Supporting Information, Figure S6).

For the positively terminated membranes a dependency of MWCO on the alkyl chain length can be observed as well. A MWCO of 260 ± 2 Da for Me-QP4VP, to 250 ± 2 Da for Et-QP4VP and 229 ± 2 Da for Pr-QP4VP is measured. All the positively terminated membranes appear to be defect-free (Supporting Information, Figure S5). The positively terminated membranes consistently show a lower MWCO than the negatively terminated membranes, indicating that these membranes are denser. The extra PDADMAC layer is likely to have led to an elimination of the defects. The denser nature of the positively terminated membranes is accordance with the permeability results. A connection between these properties is typical, and is commonly referred to as the permeability-selectivity trade-off.^39^ It occurs because a dense layer, with a low degree of swelling, promotes higher selectivity, whereas an open layer enhances higher permeability. The clear trend in permeability and MWCO for both negatively and positively terminated QP4VPs confirms our hypothesis that the introduction of some hydrophobicity in a PEM increases its density.

The PDADMAC membranes have a higher MWCO than all other defect-free membranes, which indicates that PDADMAC/PSS is less selective than other membranes presented, even though its permeability is within the range of the other defect-free membranes. The observed MWCO’s for both negatively and positively terminated Et-QP4VP and Pr-QP4VP are very low. The dNF40, the densest commercially available PEM membrane module has a MWCO of 400 Da at a permeability of 6.1 ± 0.1 L m^–2^ h^–1^ bar^–1^,^40^ showing that the QP4VP membranes clearly outperform commercial modules. Moreover, the QP4VP membranes have a clearly lower MWCO than commonly used PEM membranes in academia which were coated at similar conditions, such as a MWCO of 267 Da for poly(allylamine hydrochloric acid) (PAH)/PSS membranes.^41^ The Pr-QP4VP membranes have a MWCO comparable to negatively terminated poly(vinylamine) (PVA)/PSS (235 Da) and poly(ethylenimine) (PEI)/PSS (239 Da) membranes coated at similar conditions,^15,41^ which are among the densest multilayers used for PEM membranes. The permeability of the negatively (7.5 ± 0.5 L m^–2^ h^–1^ bar^–1^) and positively terminated (6.2 ± 0.5 L m^–2^ h^–1^ bar^–1^) Pr-QP4VP membranes is in the same range as PEI/PSS (6.5 ± 0.3 L m^–2^ h^–1^ bar^–1^) and lower than PVA/PSS (9.9 ± 0.1 L m^–2^ h^–1^ bar^–1^). However, PAH and PVA contain primary amine groups and PEI contains primary, secondary, and tertiary amine groups, making them sensitive to pH changes and hypochlorite degradation. Instead, Pr-QP4Vp contains only quaternary pyridinium groups. The MWCO experiments demonstrate that hydrophobic interactions can be used to create dense PEM membranes, with chemically stable polyelectrolytes.

In order to get an impression of the chemical stability of the QP4VPs, their integrity after the exposure to sodium hypochlorite (NaOCl) was tested (Supporting Information, Figure S7) and evaluated visually. While PAH shows signs of degradation within 2 min, PDADMAC and the QP4VPs do not show signs of degradation even after 23 h. More extensive testing should be done to get a more quantitative understanding of the chemical stability of QP4VP, such as monitoring the permeability over time with the exposure to NaOCl.^26^ Nevertheless, these initial screening results on chemical stability of QP4VPs in comparison with PAH are highly promising.

### 3.5 Salt Retention

Next, the retention of different single-component salt solutions was measured. For this, salts consisting of combinations of monovalent and divalent ions were used (NaCl, Na_2_SO_4_, MgCl_2_ and MgSO_4_). This includes salts where both ions have the same valency (symmetric salts; NaCl, MgSO_4_) and salts where both ions have a different valency (asymmetric salts; Na_2_SO_4_, MgCl_2_). This combination of salts can give an indication of the ion exclusion mechanisms which play a role in the membrane.

**Figure 5:**
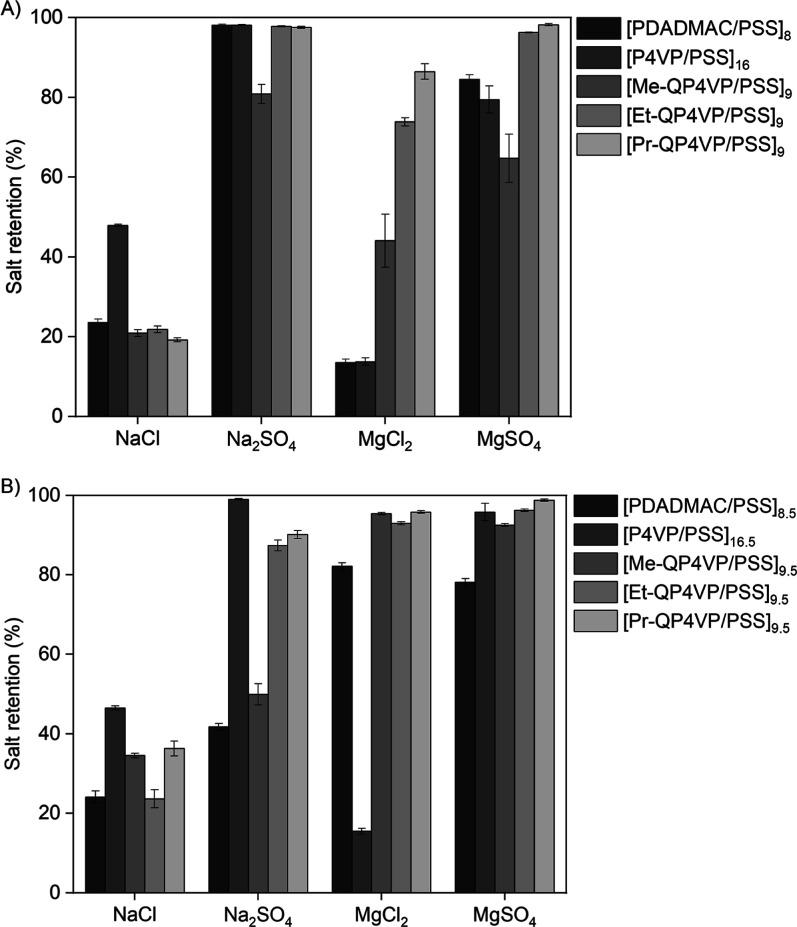
Retention of single salt solutions of (A) negatively and (B) positively terminated membranes. Error bars represent the 95% confidence interval (*n* = 4).

For the negatively terminated P4VP membrane, the sequence of ion retention is Na_2_SO_4_ (98.1 ± 0.1%) > MgSO_4_ (79.4 ± 3.4%) > NaCl (47.9 ± 0.3%) > MgCl_2_ (13.7 ± 0.9%) (Figure 5A). Such a retention pattern, where there is a big difference in the retention of the asymmetric salts is an indication for Donnan exclusion. Donnan exclusion takes place at the surface of the membrane. Here, the charged surface excludes ions with the same charge sign, while allowing ions with an opposite charge sign to pass. This effect is especially strong for divalent ions. A higher retention of Na_2_SO_4_ than MgCl_2_ therefore indicates a negatively charged membrane surface, which is in line with these membranes being terminated with a polyanion. A similar trend can be observed for the negatively charged PDADMAC membrane.

For the negatively terminated QP4VP membranes, the retention of Na_2_SO_4_ always exceeds the retention of MgCl_2_. However, the difference in retention between these two salts decreases with increasing alkyl chain length. For Me-QP4VP, Na_2_SO_4_ (80.8 ± 2.4%) and MgCl_2_ (44.1 ± 6.8%) show a substantial difference in retention. The difference between the retention of Na_2_SO_4_ and MgCl_2_ becomes smaller for Et-QP4VP (97.8 ± 0.1% and 73.8 ± 1.0%, respectively) and decreases even further for Pr-QP4VP (Na_2_SO_4_: 97.6 ± 0.3%, MgCl_2_: 86.5 ± 2.0%). This is an indication that the contribution of dielectric exclusion to the ion retention increases. Dielectric exclusion takes place in the membrane matrix and is caused by the difference in the dielectric constant of the membrane compared to that of the feed solution. In this exclusion mechanism, the ion retention is mainly determined by the valency of the ions, rather than the charge sign of the ion, such that all salts with divalent ions are retained. A higher contribution of dielectric exclusion is indicative of a denser membrane.^34^ This is in line with the permeability and MWCO measurements. It is important to note that the salt retention of the Me-QP4VP membranes may be slightly underestimated due to the presence of defects in these membranes.

The positively terminated P4VP membrane has a salt retention pattern that is very similar to the negatively terminated P4VP membrane (Figure 5B). Again, the sequence of ion retention is Na_2_SO_4_ (99.0 ± 0.2%) > MgSO_4_ (95.8 ± 2.2%) > NaCl (46.5 ± 0.5%) > MgCl_2_ (15.5 ± 0.7%). This is remarkable because, in a Donnan exclusion membrane with a positively charged layer, MgCl_2_ retention would be expected to exceed Na_2_SO_4_ retention, as seen in the positively terminated PDADMAC membrane. In contrast, the observed salt retention pattern would normally occur for a negatively terminated membrane. The discrepancy can be explained by the influence of pH. As mentioned before, the P4VP layers were coated at pH 1.5 to dissolve the P4VP, while these salt retentions are measured at neutral pH. It has been noted before that the salt retention of PEM membranes with one weak and one strong polyelectrolyte can be highly affected by the feed pH,^42^ as (de)protonation of the polyelectrolyte can take place. Thus, the lack of positive charge is likely resulting from partial deprotonation of P4VP at neutral pH, leading to a membrane with a surplus of negative charge. This explains why the retention of Na_2_SO_4_ far exceeds the MgCl_2_ retention, and why no strong odd–even effect^9^ can be seen for these membranes. Nevertheless, the surface chemistry of these membranes is likely to be still different. Hence, this deprotonation mechanism does offer opportunities to further select for desired membrane properties.

For the positively terminated QP4VPs, the MgCl_2_ retention exceeds the Na_2_SO_4_ retention, indicating a positively charged surface. However, in the case of Et-QP4VP and Pr-QP4VP these differences are minor, indicating that dielectric exclusion again plays a major role in the ion retention of these membranes. Once more, this is in line with the dense nature of these membranes which was indicated by the permeability and MWCO experiments. Overall, the salt retention experiments confirm that hydrophobic interactions can be used to create dense PEM membranes. This is most likely because of less hydration (swelling) in these membranes.

### 3.6 OMP Retention

**Figure 6:**
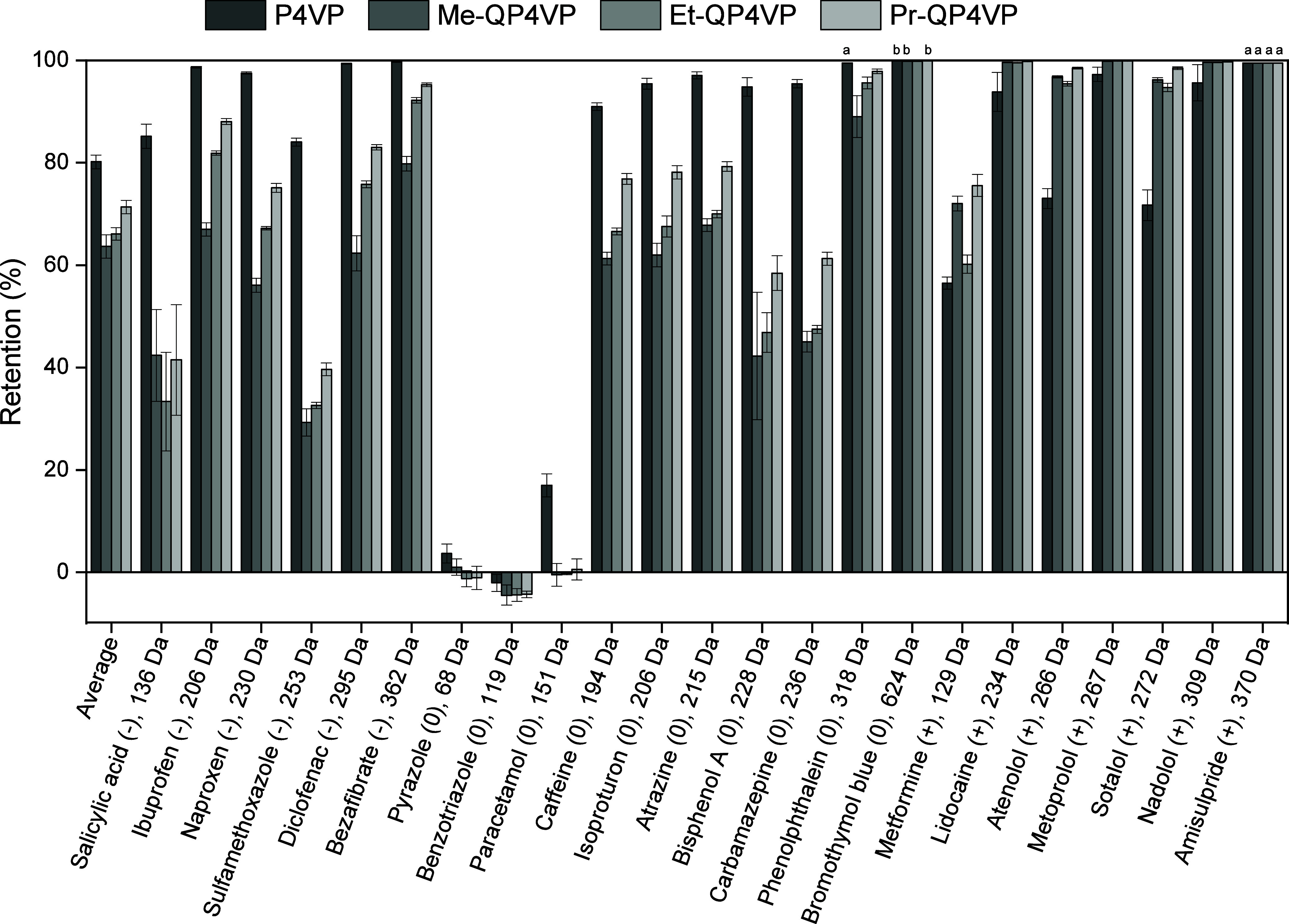
Organic micropollutant (OMP) retention of positively terminated polyelectrolyte multilayer (PEM) membranes. In some cases, the OMP retention was above the quantification limit. This is indicated with a marker (quantification limits: *a* = 99.5%, *b* = 99.9%). The displayed retention is the quantification limit, actual OMP retention may be higher. Error bars represent the 95% confidence interval (*n* = 4).

Lastly, the OMP retention of the positively terminated membranes was measured. Positively terminated membranes were selected as these were the densest membranes, which were expected to give the highest OMP retention. A feed mixture consisting of model OMPs was used for determination of OMP retention. The model OMPs were selected to represent a wide range of properties (charge, molecular weight) and are used for a variety of different applications (pharmaceuticals, industrial chemicals, herbicides, pH indicators). Because of this variety in characteristics, a good overview can be obtained. The obtained OMP retentions vary wildly, from negligible retentions for small neutral OMPs (pyrazole, benzotriazole) to near full retention of amisulpride (Figure 6). A detailed overview of the obtained retentions and quantification limits can be found in the Supporting Information, Tables S1 and Table S2.

The effect of different properties of the OMPs is reflected in their retentions. OMP charge plays a substantial role in determining its retention. Substantial differences in retention can be observed between the different membranes. While the P4VP/PSS membranes show comparatively high retentions for negatively charged and neutral OMPs, The QP4VP membranes generally show higher retentions for positively charged OMPs. This difference can be explained though the difference in charge of the membranes. As could be seen with the salt retention measurements, the P4VP/PSS membranes appear to have a negative charge, facilitating retention of negatively charged OMPs. The QP4VP membranes on the other hand have a positive charge, facilitating the retention of positively charged OMPs. At time of writing, it is unclear why the P4VP membrane has such a high retention of neutral OMPs, especially since the MWCO of the P4VP membrane is substantially higher than that of the Pr-QP4VP membrane. This does show that applicability of P4VP/PSS for OMP removal is worthy of further investigation, although there is also a possibility that the P4VP PEM layers are thicker than expected and therefore adsorb more OMPs than the QP4VP layers, leading to higher apparent retentions still after 28 h of filtration.

Generally, higher retentions are observed for larger OMPs, indicating a contribution of size exclusion to their retention. One notable deviation from this trend is sulfamethoxazole (253 Da), which shows a lower retention than smaller negatively charged OMPs such as ibuprofen and naproxen. While sulfamethoxazole is grouped with the negatively charged compounds, it has a p*K*_a_ of 6.16,^43^ meaning that it is only partially charged at the pH of the feed mixture.

The contribution of size exclusion is also reflected in the OMP retention trend for the different QP4VP membranes. Generally, the OMP retention increases upon increase of the alkyl chain length, with Me-QP4VP/PSS having lower retentions than Pr-QP4VP/PSS. This is in line with the MWCO data, which demonstrated that Pr-QP4VP is a denser membrane. It clearly shows that increasing hydrophobic interactions is a viable method for increasing OMP retention, and that especially high retentions can be obtained for positively charged OMPs.

The OMP retention of the P4VP and QP4VP membranes was compared with [PDADMAC/PSS]_8_ membranes (Supporting Information, Figure S8), the most commonly studied PEM system based on quaternary amines. The average OMP retentions of the P4VP (80.2 ± 1.3%), Me-QP4VP (63.7 ± 2.3%), Et-QP4VP (66.1 ± 1.2%) and Pr-QP4VP (71.4 ± 1.3%) membranes are much higher than the average OMP retention of the PDADMAC membrane (39.9 ± 2.2%). Although the PDADMAC membrane is negatively terminated while the P4VP and QP4VP membranes are positively terminated, the OMP mix consists of positive, neutral, and negative compounds. Therefore, the membrane surface charge cannot account for the substantial difference in average retention observed. Thus, the OMP retention of the P4VP and QP4VP membranes is a considerable improvement with regards to the PDADMAC membrane.

## 4 Conclusion and Outlook

In this study, the effect of hydrophobic interactions on the properties of PEM membranes was evaluated. Hydrophobicity was introduced in a controlled manner through the alkylation of P4VP with alkyl chains of increasing chain length. Our results clearly show that more hydrophobic polycations can yield denser PEM membranes, with a lower permeability and a lower MWCO. Membranes with a MWCO as low as 230 Da could be created. The membranes are substantially denser and more selective than PDADMAC/PSS membranes. The more hydrophobic polycations give a higher salt retention and a switch from Donnan exclusion to dielectric exclusion as the dominant ion retention mechanism with increasing chain length. The OMP retention increases upon increasing chain length as well. Besides that, the quaternary pyridinium group will bestow these membranes with a higher chemical stability than other dense PEM membranes based on primary, secondary, or tertiary amines.

Future research could optimize the use of hydrophobic interactions. Potentially even denser membranes could be fabricated by further increasing the alkyl chain length. Successful alkylation of P4VP has been reported with alkyl chains as long as 12 carbons in length,^44^ showing potential for elaboration of this concept. Besides that, a more systematic investigation of the role of other intermolecular interactions could contribute to a more rational design of PEM membranes.

In conclusion, our study highlights the importance of hydrophobic interactions for the construction of PEM membranes. While the importance of hydrophobic interactions in the construction of PEMs has been well-established,^16^ its implications for the properties of PEM membranes have thus far been overlooked. These insights can be particularly useful for the construction of dense PEM membranes for the removal of OMPs from wastewaters.

## Supporting Information Available

^1^H NMR spectra, refractometry data, FE-SEM images, molecular weight cutoff sieving curves, additional permeability and MWCO data for [Me-QP4VP/PSS]_9_, [Me-QP4VP/PSS]_9.5_ and [Me-QP4VP/PSS]_10_, photos of polyelectrolyte/NaOCl solutions, HPLC-MS signal-to-noise ratio and data for OMP retention including [PDADMAC/PSS]_8_ membranes (PDF)
