# Supplementary material for: Highly Selective Polyelectrolyte Multilayer Membranes Through Hydrophobic Interactions
Source: ACS Appl Mater Interfaces. 2025 Mar 27;17(14):21725–35. doi: 10.1021/acsami.4c20150 (PMC11986897; doi:10.1021/acsami.4c20150)
Supplement: Supplementary file 1 — am4c20150_si_001.pdf [file am4c20150_si_001.pdf]

Supporting information

Highly selective polyelectrolyte multilayer membranes through hydrophobic interactions

Wendy A. Jonkers[a], Maxime Precheur[a], J. Roberto Andrade[a], Wiebe M. de Vos[a] and Esra te Brinke[a*]

[a] Membrane Science and Technology, University of Twente, MESA+ Institute for Nanotechnology, P.O. Box 217, 7500 AE, Enschede, the Netherlands

[*]Corresponding author, email: e.tebrinke@utwente.nl

1. Analysis of synthesized QP4VPs

Hydrophobic polycations had to be synthesized as they were not commercially available. This was done by quaternization of P4VP with alkyl halides. The reaction products were analyzed with ^{1}H NMR to evaluate the purity of the reaction products (Figure S1). Spectra are normalized with respect to the D_2O peak at shift 4.7900. Peak integration confirmed that full quaternization could be obtained with all alkyl halides in DMSO.

This spectrum also shows the synthesis of 75%Me-QP4VP (P4VP with 75% of monomers being quaternized with methyl, and 25% being unquaternized). For the synthesis of this compound, 2.00 g of P4VP was dissolved in water. Subsequently, a 500% molar excess of iodomethane was added. The solution was stirred overnight at room temperature. After the reaction was completed, the reaction mixture was purged with N_2 for four hours. Finally, the mixture was freeze dried to obtain the dried product, which was redissolved in Milli-Q water. **75%Me-QP4VP**: ^{1}H NMR (D_2O, 400 MHz) δ 1.73 (br, 3H), 4.22 (br, 2.3H), 6.66 (br, 0.5H), 7.36 (br, 1.5H), 8.03 (br, 0.5H), 8.46 (br, 1.5H).

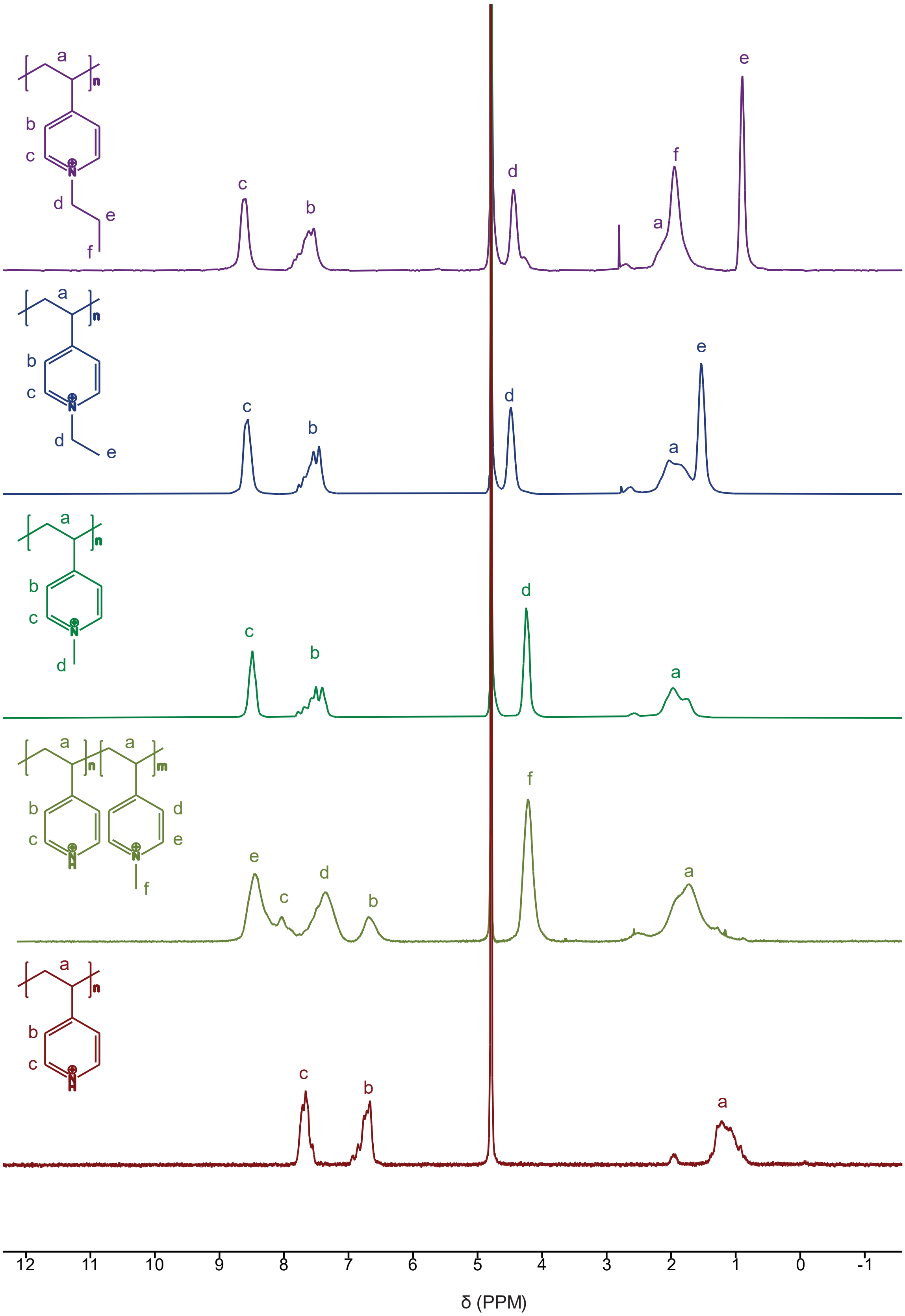

Figure S1: ^{1}H NMR spectra of P4VP, 75%Me-QP4VP, Me-QP4VP, Et-QP4VP and Pr-QP4VP. For P4VP, a mixture of D_2O and DCl was used as a solvent, the quaternized polyelectrolytes were dissolved in D_2O only. Spectra are plotted with an arbitrary y-axis offset to show the differences between the different plots.

2. Refractometry

In order to be able to calculate the Q-factor, which was used in reflectometry, the dn/dc of each polyelectrolyte was determined. For this, samples of 0, 0.1, 0.5, 1, 5 and 10 g L^{-1} polyelectrolyte in Milli-Q water containing 50 mM NaCl were prepared. The pH of P4VP was adjusted to 1.5. The refractive index at 632.8 nm was determined with a Schmidt+Haensch ATR-lambda refractometer. The dn/dc of each polyelectrolyte was obtained by determining the slope of a linear fit of the refractive index over the polymer concentration (Figure S2). This yielded dn/dc values for P4VP (0.2933 mL g^{-1}), Me-QP4VP (0.2053 mL g^{-1}), Et-QP4VP (0.1851 mL g^{-1}) and Pr-QP4VP (0.2142 mL g^{-1}).

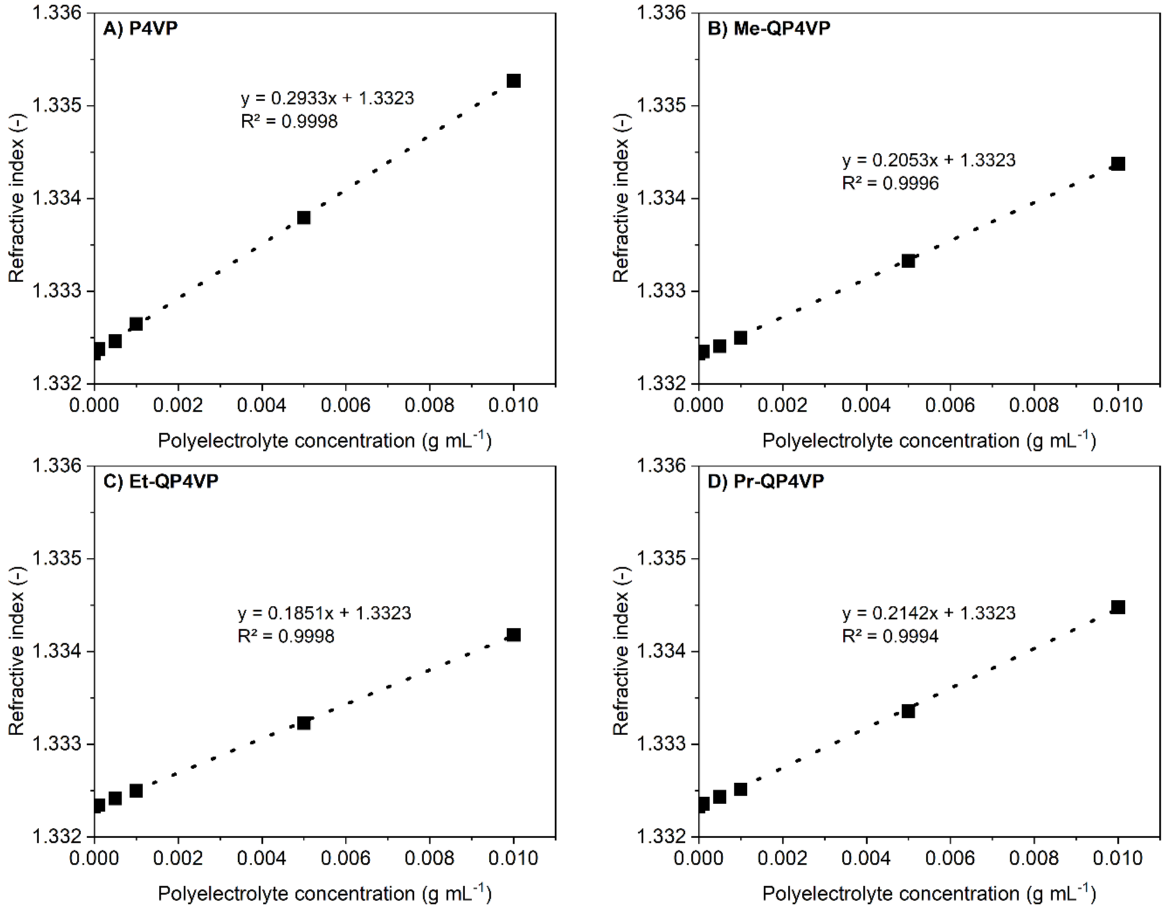

Figure S2: Refractive index increment (dn/dc) for the polycations used in this paper. Each polycation is dissolved in Milli-Q water with 50 mM NaCl. The error bars, depicting the 95% confidence intervals (n=3), overlap with the data markers owing to the narrow range of the confidence intervals. A) P4VP, B) Me-QP4VP, C) Et-QP4VP and D) Pr-QP4VP.

3. FE-SEM

Field emission scanning electron microscopy (FE-SEM) images were made to assess the impact of the addition of alkyl chains on the structure of the membrane surface. As preparation, the coated membranes were immersed in isopropanol for 30 minutes and then dipped in liquid N_2. The membranes for the cross-section images were broken and mounted onto a sample holder with carbon tape. The membranes for the inner surface images were cut and mounted on sample holders with hot glue. To remove dust from the membranes, the sample holders were placed in a vacuum oven overnight at 30 °C. Then, a 5 nm Pt/Pd coating was applied onto the samples with a Quorum Q150T ES sputter coater at 0.5 bars of Ag pressure. The samples were measured on a JEOL JSM-7610F FE-SEM at 100,000 times magnification.

When comparing the cross-section of the support membranes (Figure S3) to the coated membranes, it can be seen that the PEM layer is extremely thin, in the order of tens of nanometers. This is in line with the reflectometry results. No major differences between the cross-sections of the coated membranes can be observed. The inner surface image of the support reveals distinct lines, which result from the production process. These lines become less noticeable after the membranes are coated, indicating successful coating. However, their continued visibility suggests that the coating is very thin.

Membrane | Cross-section | Inner surface

Support

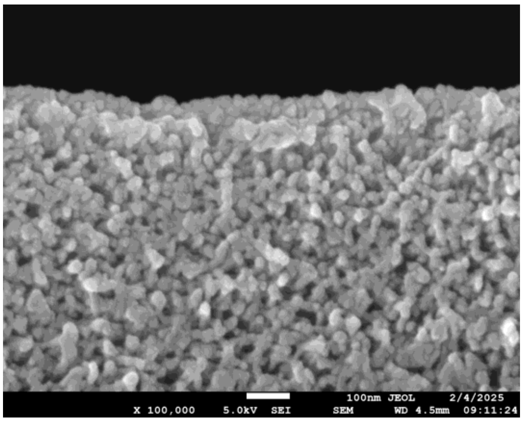

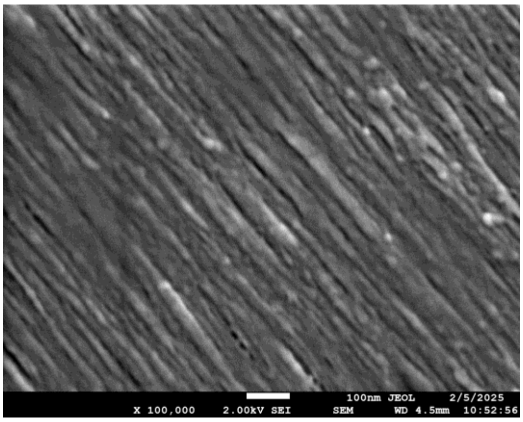

[PDADMAC/PSS]$_8$

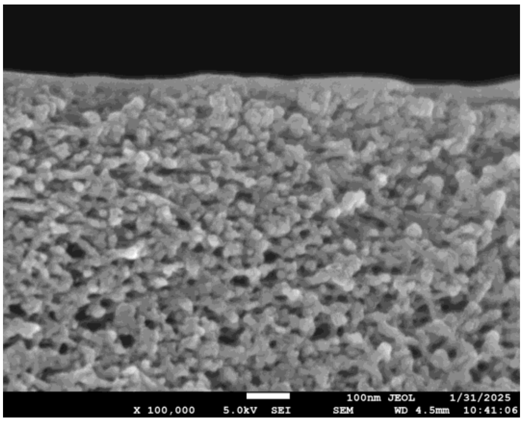

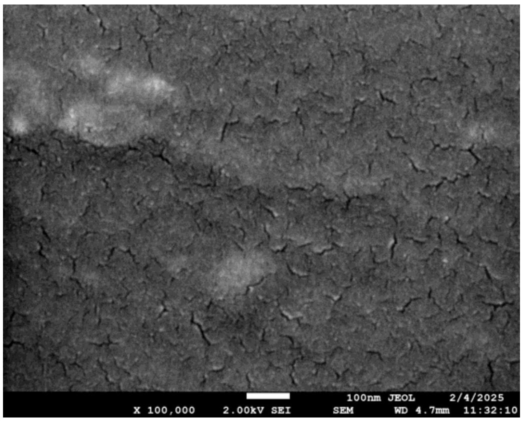

[PDADMAC/PSS]$_{8.5}$

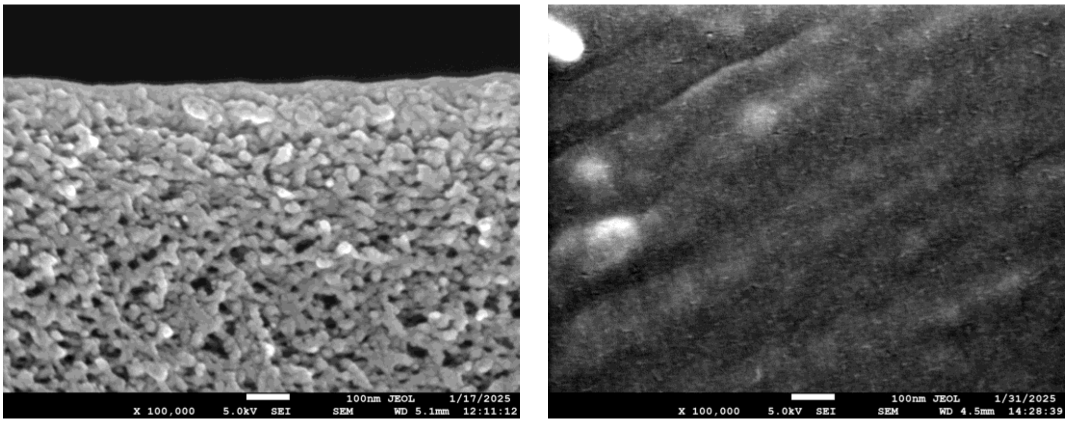

[P4VP/PSS]$_{16}$

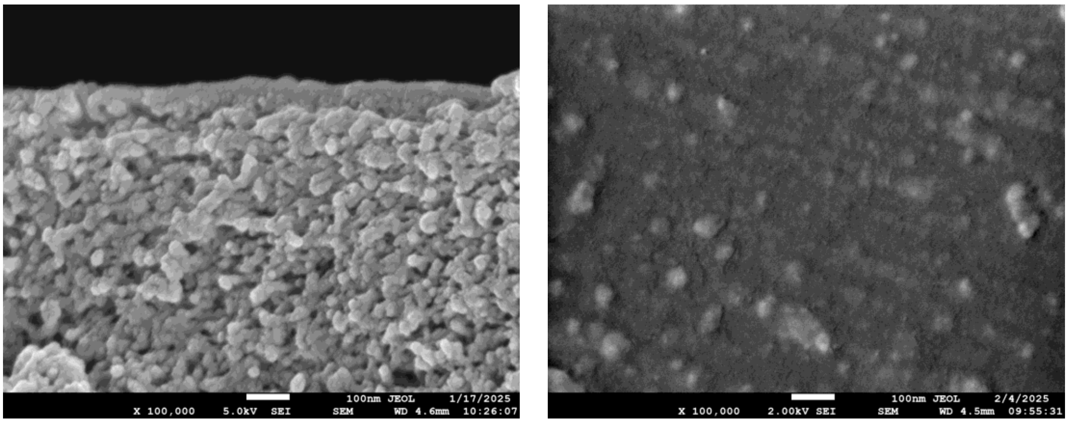

[P4VP/PSS]$_{16.5}$

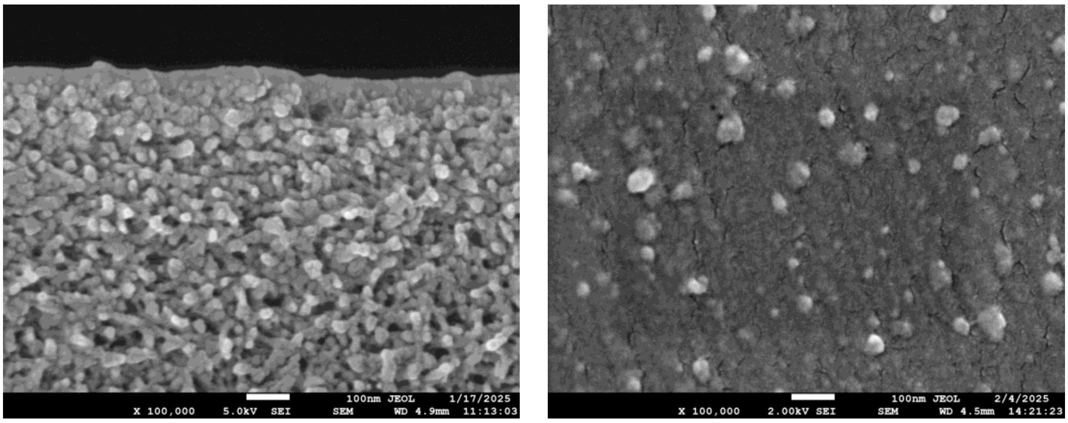

[Me-QP4VP/PSS]$_{9}$

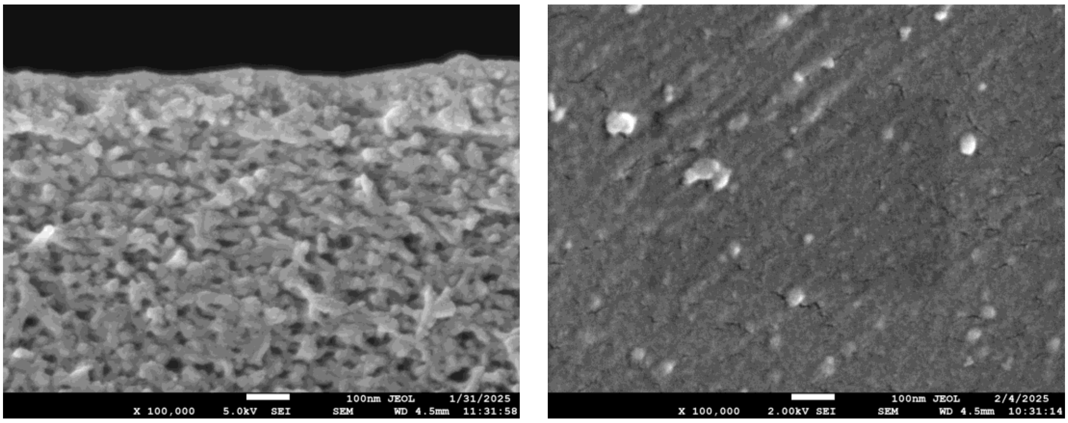

[Me-QP4VP/PSS]$_{9.5}$

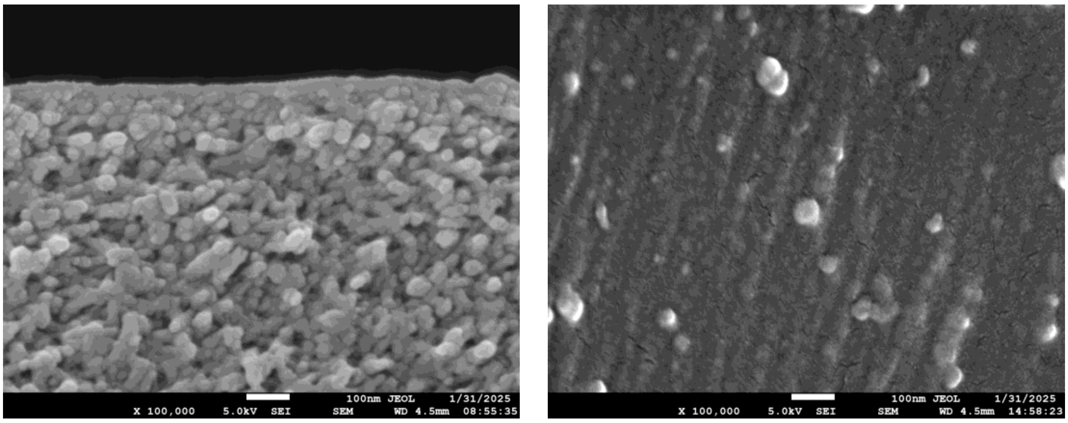

[Et-QP4VP/PSS]$_9$

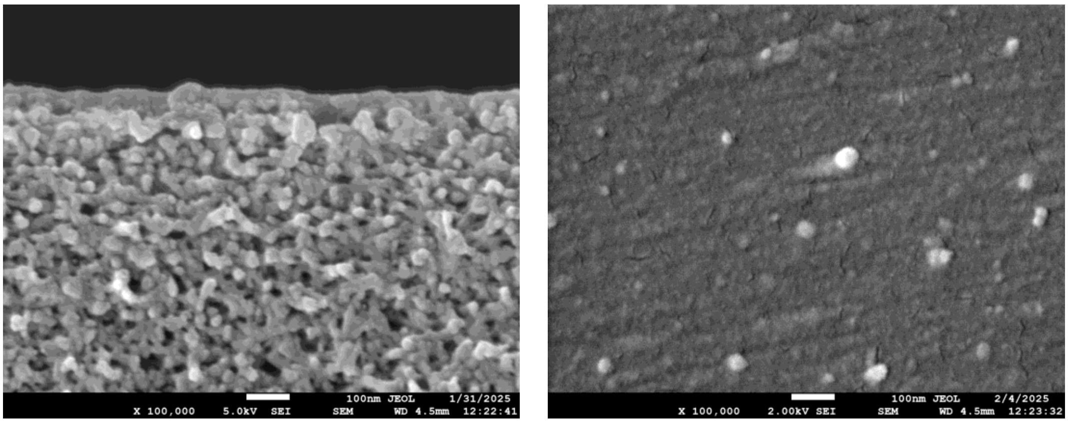

[Et-QP4VP/PSS]$_{9.5}$

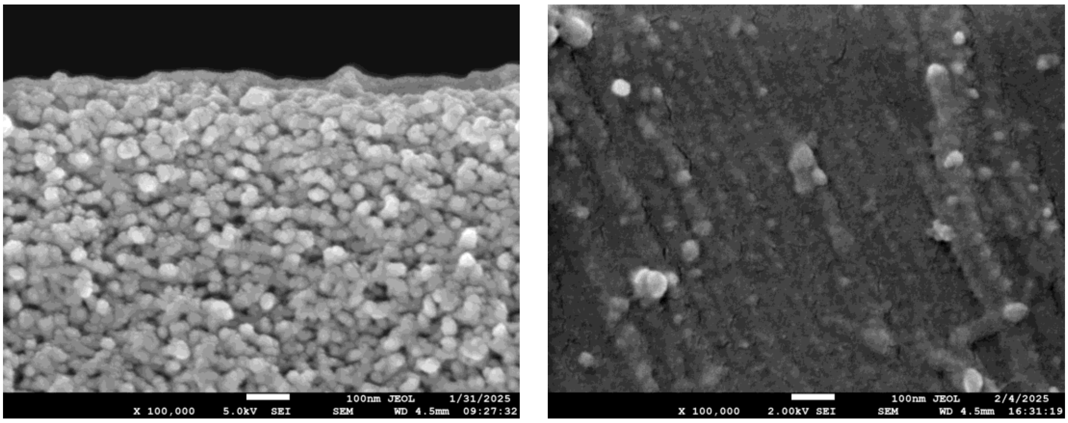

[Pr-QP4VP/PSS]$_9$

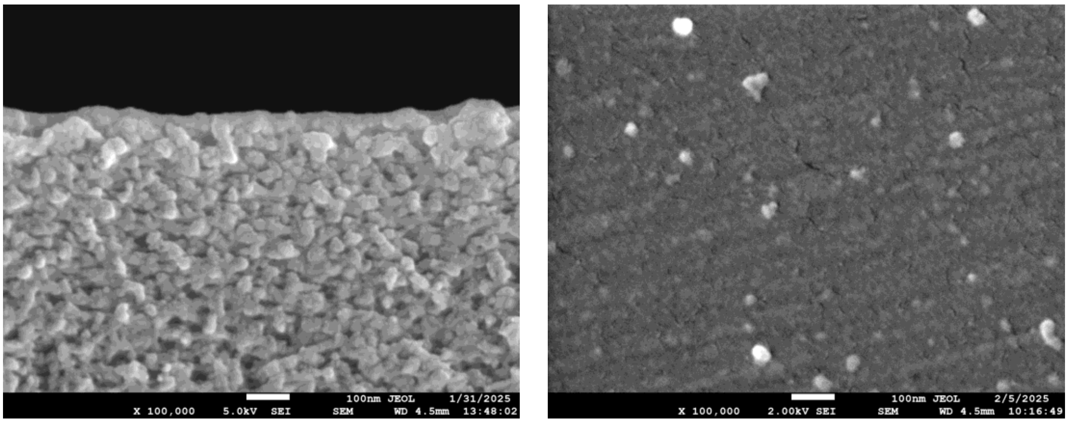

$[Pr\text{-}QP4VP/PSS]_{9.5}$

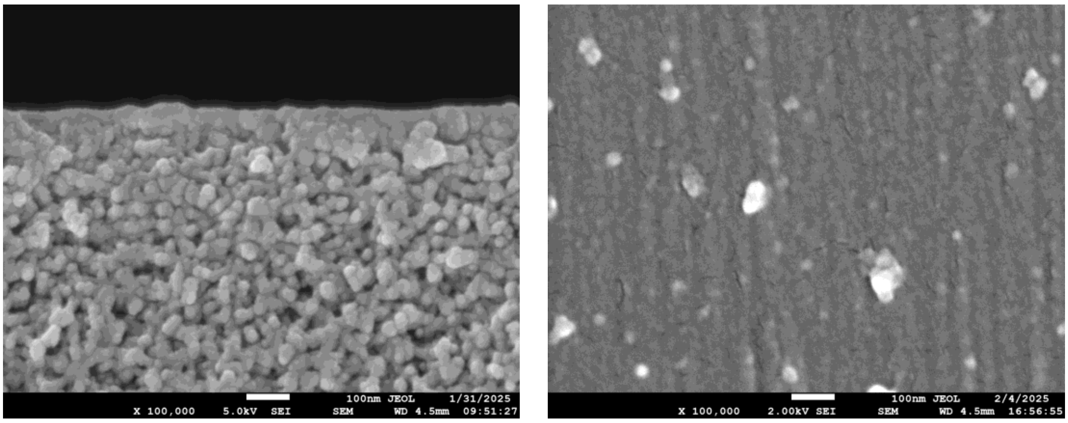

Figure S3: Field emission scanning electron microscopy (FE-SEM) images of the cross-section and the inner surface of the membranes presented in this study.

4. Molecular weight cut-off

Based on the PEG retention tests to determine the MWCO of the membranes, sieving curves were plotted (Figure S4, Figure S5). The sieving coefficient is plotted on the y-as. It is a dimensionless quantity that represents the ratio of the concentration of a compound with a certain molecular weight in the permeate divided by concentration of the compound in the feed. The 90% MWCO represents a sieving coefficient of 0.1 and is represented in the graph by a data marker.

The sieving curves can give more information about potential defects in the membranes. For defect-free membranes, the sieving coefficient is expected to go to 0 for large molecular weights, as can be seen for all positively terminated membranes (Figure S5). If the sieving coefficient does not smoothly approach zero for large molecular weight compounds, this is an indication for defects in the membrane. Indeed, this can be seen for the negatively terminated P4VP and Me-QP4VP membranes (Figure S4). In addition, it can be seen that the determined 90% MWCO varies substantially, which corresponds to the large error bars in Figure 4B (main text).

Negatively terminated membranes

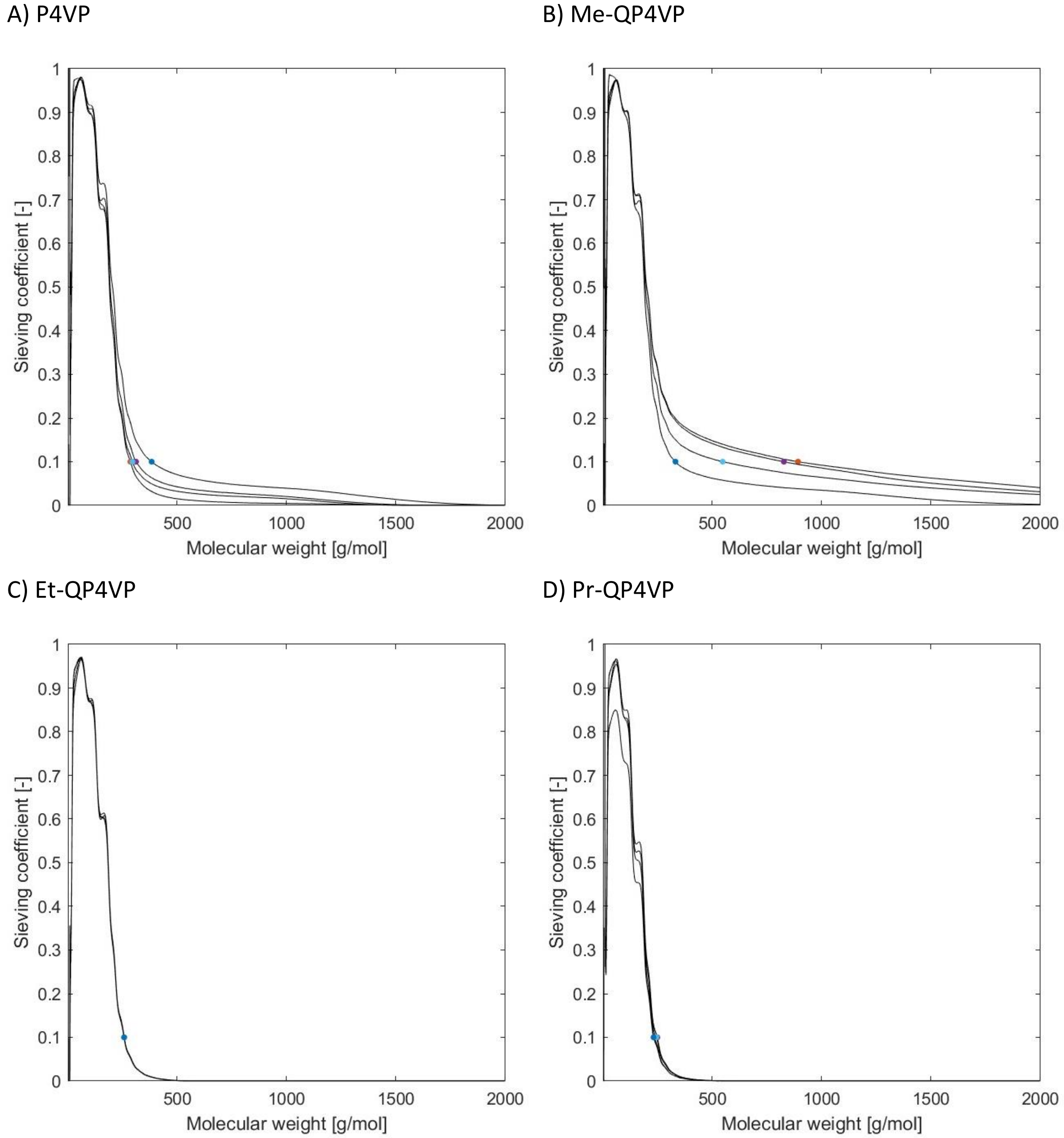

Figure S4: Sieving curves of negatively terminated membranes. The 90% MWCO is indicated with a data marker. A) P4VP, B) Me-QP4VP, C) Et-QP4VP and D) Pr-QP4VP. P4VP and Me-QP4VP show clear defects (data markers do not overlap, sieving coefficient does not quickly approach 0).

Positively terminated membranes

A) P4VP

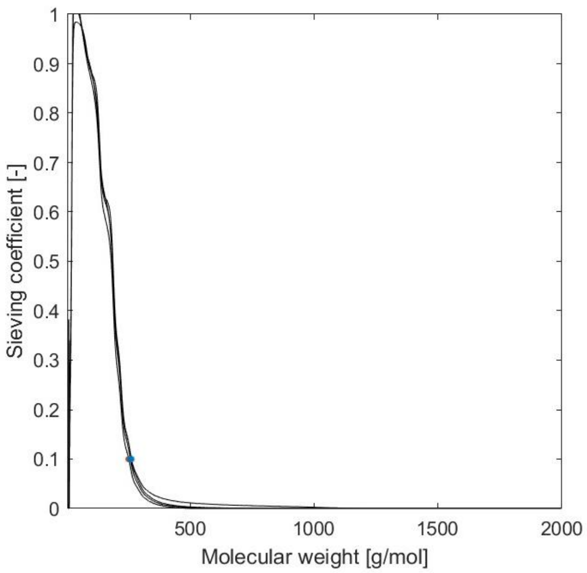

B) Me-QP4VP

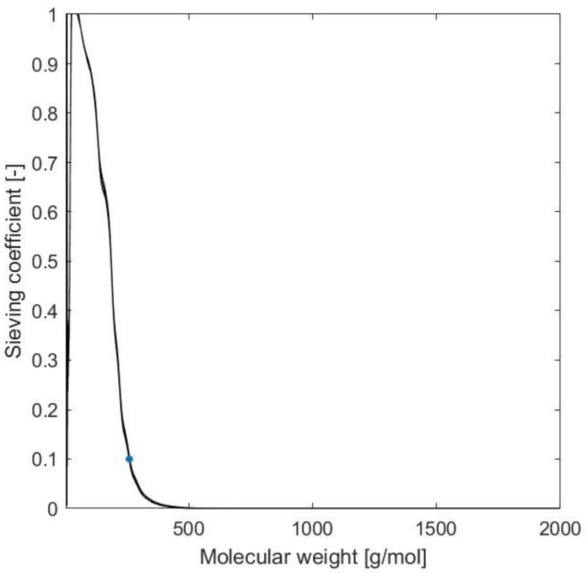

C) Et-QP4VP

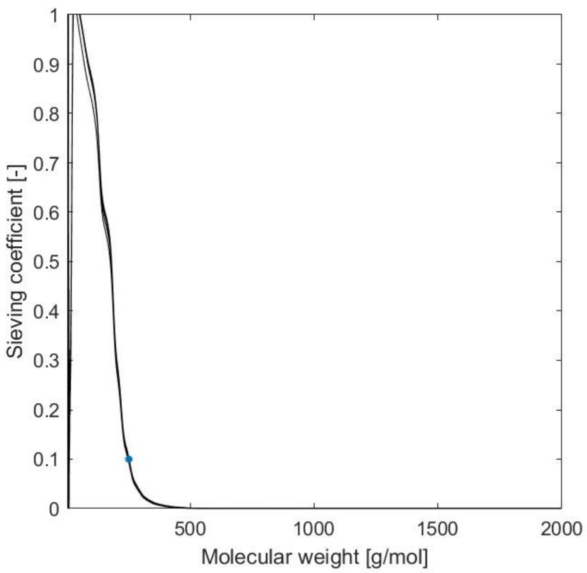

D) Pr-QP4VP

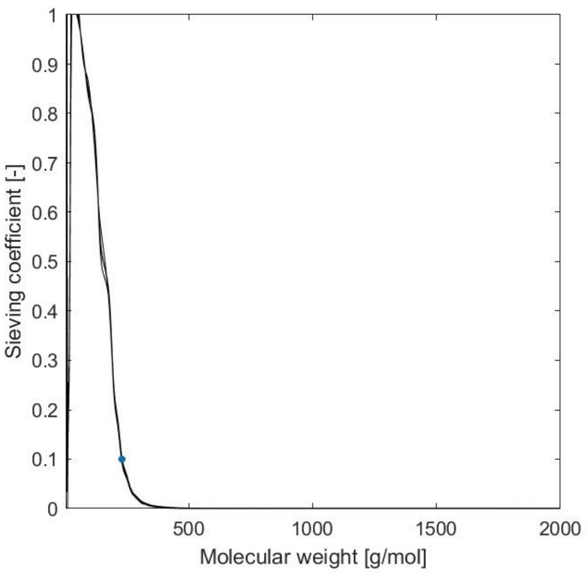

Figure S5: Sieving curves of positively terminated membranes. The 90% MWCO is indicated with a data marker. A) P4VP, B) Me-QP4VP, C) Et-QP4VP and D) Pr-QP4VP.

5. Defects

Defects in a PEM membrane can occur when the pores of the support membrane have not fully been closed. By coating more layers on top of the support membrane, the membrane can transition from a pore-dominated regime to a layer-dominated regime [1]. To assess if the defects in the [Me-QP4VP/PSS]$_9$ membrane can be diminished by coating more layers on top of the membrane, a new set of [Me-QP4VP/PSS]$_9$ was selected and extra layers were coated on top of these membranes, to obtain [Me-QP4VP/PSS]$_{9.5}$ and [Me-QP4VP/PSS]$_{10}$ membranes. Figure S6A demonstrates that the permeability substantially decreases as more layers are applied. Additionally, Figure S6B shows a reduction in average MWCO and the decrease of the size of the error bars with the coating of extra layers. The average MWCO of [Me-QP4VP/PSS]$_9$ is substantially lower than that shown in Figure 4B, highlighting a significant variation in the [Me-QP4VP/PSS]$_9$ membrane population. The sieving curve in Figure S6C again indicates defects for one of the [Me-QP4VP/PSS]$_9$ membranes. In contrast, the sieving curves for [Me-QP4VP/PSS]$_{9.5}$ and [Me-QP4VP/PSS]$_{10}$ in Figure S6D and Figure S6E demonstrate that these membranes are defect-free. This indicates that defect-free, negatively terminated Me-QP4VP/PSS membranes can be fabricated when extra layers are added.

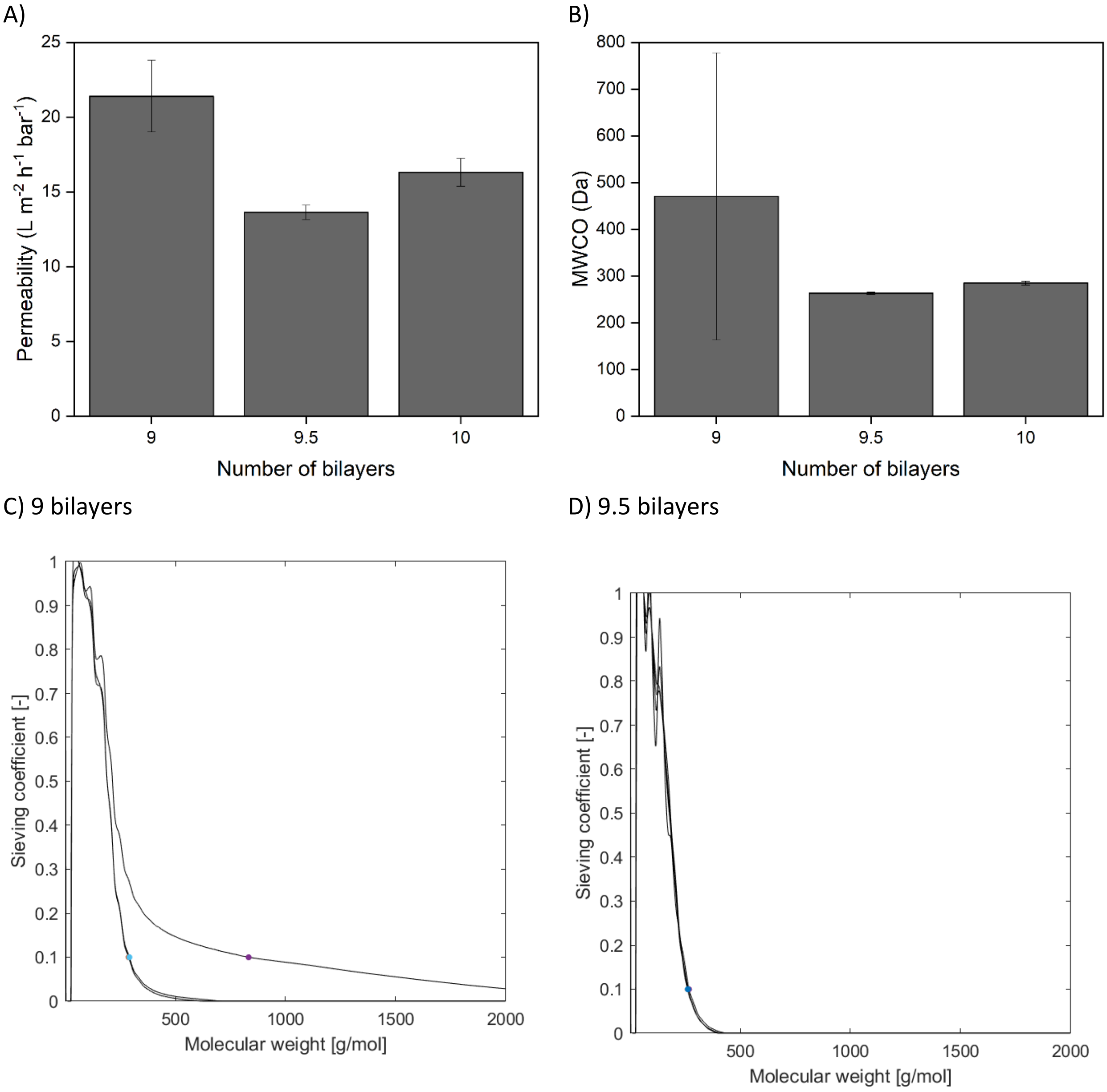

E) 10 bilayers

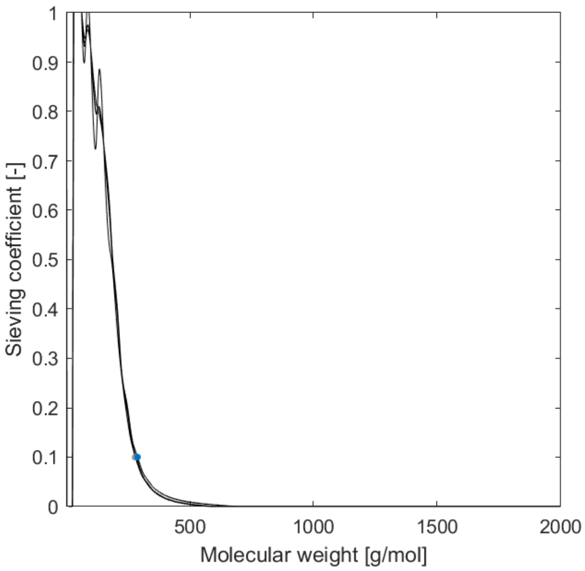

Figure S6: Performance of Me-QP4VP/PSS membranes. A) Permeability B) MWCO, C), D), E) sieving curves

6. Chemical stability

In order to get an impression of the chemical stability of the QP4VPs, their integrity after the exposure to sodium hypochlorite (NaOCl) was tested (Figure S7). For this, 3 ml 0.08% NaOCl (pH 8) was added to 3 ml 0.25 g/L polyelectrolyte (pH 8). The integrity of the samples was visually inspected. The PAH sample forms clumps within 2 minutes. This is a clear sign of degradation [2]. All the other samples remain clear, suggesting that these have a higher chemical stability than PAH.

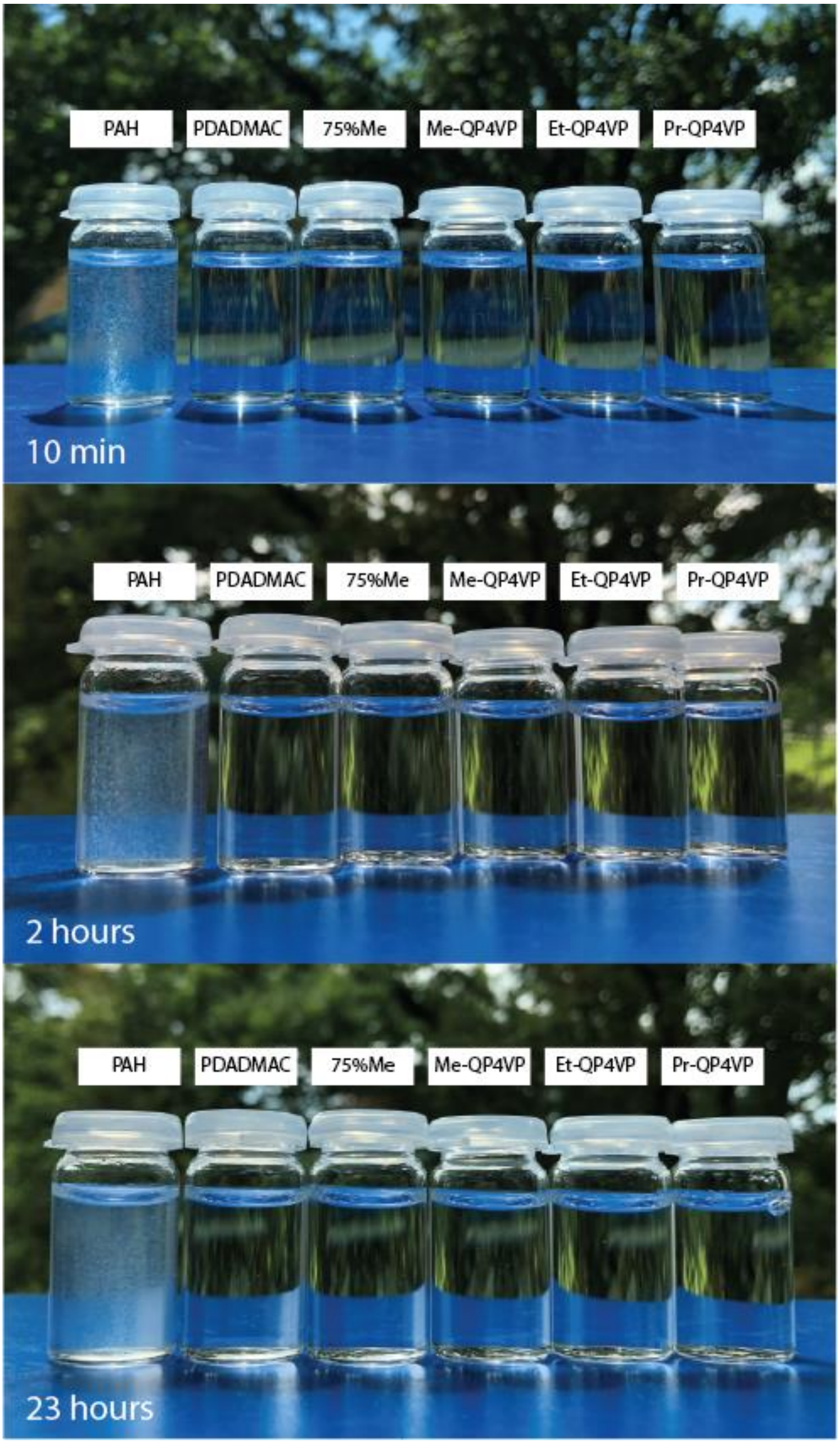

Figure S7: Visual assessment of the chemical stability of PAH, PDADMAC, 75%Me-QP4VP, Me-QP4VP, Et-QP4VP and PR-QP4VP in hypochlorite. Cloudy mixtures indicate degradation of the polyelectrolytes, and clear mixtures indicate a stable polyelectrolyte.

7. OMP retention

Table S1: Retention of the compounds Salicylic acid (SA), ibuprofen (IBU), naproxen (NPX), sulfamethoxazole (SMX), diclofenac (DIC), bezafibrate (BEZA), pyrazole (PYR), benzotriazole (BTA), paracetamol (PAR), caffeine (CAF), isoproturon (IPU), atrazine (ATR), bisphenol A (BPA), carbamazepine (CBZ), phenolphthalein (PHTH), bromothymol blue (BTB), metformin (MET), lidocaine (LIDO), atenolol (ATN), metoprolol (METO), sotalol (SOT), nadolol (NAD) and amisulpride (AMI) by $[P4VP/PSS]_{16.5}$, $[Me\text{-}QP4VP/PSS]_{9.5}$, $[Et\text{-}QP4VP/PSS]_{9.5}$, and $[Pr\text{-}QP4VP/PSS]_{9.5}$ membranes. Values highlighted in green represent retentions below the quantification limit, values highlighted in red represent retentions above the quantification limit. A, B, C and D represent replicates of the OMP retention tests performed with different membranes.

**Compound**		**SA**	**IBU**	**NPX**	**SMX**	**DIC**	**BEZA**	**PYR**	**BTA**	**PAR**	**CAF**	**IPU**	**ATR**	**BPA**	**CBZ**	**PHTH**	**BTB**	**MET**	**LIDO**	**ATN**	**METO**	**SOT**	**NAD**	**AMI**
OMP charge		(-)	(-)	(-)	(-)	(-)	(-)	0	0	0	0	0	0	0	0	0	0	(+)	(+)	(+)	(+)	(+)	(+)	(+)
OMP MW (Da)		138	206	230	253	295	362	68	119	152	194	206	215	228	236	318	624	129	234	266	267	272	309	370
Ionization mode		(-)	(-)	(-)	(+)	(+)	(+)	(+)	(+)	(+)	(+)	(+)	(+)	(-)	(+)	(+)	(+)	(+)	(+)	(+)	(+)	(+)	(+)	(+)
Quantification limit (%)		99	99.9	99.9	99	99.9	99.9	1	99.5	99.5	95	99.5	99.9	99	99.9	99.5	99.9	99.5	99.9	99.5	99.9	99.9	99.9	99.5
Retention																								
P4VP	A	86.7	98.8	97.8	84.2	99.5	99.8	4.0	-1.4	17.3	91.4	96.8	97.8	97.0	96.2	100.0	≥ 99.9	57.7	97.0	75.3	98.6	75.6	98.2	99.8
	B	87.6	98.5	97.5	84.4	99.2	99.6	6.2	-0.6	18.6	90.2	94.2	96.2	92.7	94.4	99.9	99.9	56.6	88.2	71.9	95.3	69.7	90.3	99.6
	C	84.3	98.6	97.3	82.9	99.4	99.8	2.5	-4.5	13.7	90.5	95.2	96.9	94.4	95.3	99.9	≥ 99.9	54.9	94.4	70.9	97.4	68.8	96.7	99.8
	D	82.2	98.7	97.6	84.7	99.5	99.8	2.0	-1.7	18.4	91.8	95.7	97.4	95.4	96.0	99.9	≥ 99.9	56.9	95.7	74.0	97.9	72.8	97.4	99.8
Me-QP4VP	A	52.4	67.0	56.4	25.7	61.9	80.0	3.0	-4.0	-1.5	60.3	60.5	66.3	37.8	42.9	88.9	≥ 99.9	70.0	99.6	96.6	99.8	95.8	99.6	99.8
	B	34.9	67.6	56.7	29.5	61.8	81.2	1.5	-2.6	-2.7	62.6	59.5	67.8	31.4	45.8	83.5	≥ 99.9	72.0	99.6	96.8	99.8	95.8	99.7	99.9
	C	34.3	65.2	54.0	29.7	58.6	77.8	-0.9	-7.3	-0.4	60.2	63.8	67.8	39.3	44.0	90.2	≥ 99.9	73.2	99.6	97.1	99.8	96.6	99.6	99.8
	D	48.0	68.2	57.2	32.3	67.1	80.4	0.6	-3.8	2.7	62.2	64.1	69.4	60.6	47.5	93.5	≥ 99.9	73.1	99.7	96.9	99.8	96.5	99.7	99.9
Et-QP4VP	A	38.4	82.0	67.6	32.3	75.8	92.2	-1.9	-2.5	-0.1	66.8	70.3	70.9	51.9	47.2	96.0	99.8	60.2	99.6	95.7	99.9	95.2	99.6	99.9
	B	22.4	82.3	67.4	32.0	76.4	92.8	-0.9	-5.0	-0.6	67.0	65.6	69.2	43.7	47.6	94.6	99.9	57.7	99.5	94.8	99.8	93.5	99.5	99.9
	C	28.3	82.1	67.2	33.4	76.2	92.3	-2.9	-5.3	-0.1	67.1	66.3	70.1	43.7	48.5	94.8	99.8	61.7	99.6	95.6	99.9	94.7	99.6	99.9
	D	44.3	81.2	66.8	32.7	74.8	91.5	0.8	-4.9	-0.2	65.6	68.3	69.8	48.3	46.6	97.1	99.8	61.3	99.6	95.8	99.9	95.3	99.6	99.9
Pr-QP4VP	A	48.8	88.8	76.0	40.4	83.8	95.6	1.5	-4.1	3.6	78.2	79.1	80.6	59.2	62.9	98.2	100.0	77.0	99.9	98.7	99.9	98.8	99.8	99.9
	B	28.8	88.1	75.3	38.4	82.7	94.9	-0.2	-4.9	-1.4	76.3	79.2	79.4	60.7	60.9	97.6	99.8	75.7	99.7	98.4	99.9	98.5	99.7	99.8
	C	52.4	87.3	73.9	41.0	82.6	95.0	-1.8	-4.7	0.3	77.2	76.3	78.6	53.4	61.5	97.4	100.0	77.1	99.8	98.6	99.9	98.6	99.8	99.9
	D	36.1	88.2	75.2	38.8	83.1	95.5	-3.9	-3.5	-0.1	75.7	78.1	78.5	60.6	59.8	98.3	100.0	72.4	99.7	98.3	99.8	98.2	99.7	99.8

Table S2: Signal-to-noise ratio (STNR) of (diluted) feed samples, used for the calibration, and permeates for the HPLC-MS measurements. A STNR ≥ 10.0 is considered as the quantification limit. For the calibration series, all samples with an STNR ≥10 have been highlighted in green. A, B, C and D represent replicates of the OMP retention tests performed with different membranes.

Sample		SA	IBU	NPX	SMX	DIC	BEZA	PYR	BTA	PAR	CAF	IPU	ATR	BPA	CBZ	PHTH	BTB	MET	LIDO	ATN	METO	SOT	NAD	AMI
Ionization mode		(-)	(-)	(-)	(+)	(+)	(+)	(+)	(+)	(+)	(+)	(+)	(+)	(-)	(+)	(+)	(+)	(+)	(+)	(+)	(+)	(+)	(+)	(+)
Feed	1000x	1.9	21.7	27.3	2.3	11.6	16.4	5	2.9	3.3	2.6	3.7	15.5	5.2	51.3	7.2	153.6	7.3	18	8.8	12.5	10.4	25	13.9
	200x	9.4	94.4	24.8	9.7	50.8	36.1	4.9	14.6	20.4	4.7	28.1	28.9	8.8	137.4	25.1	88.2	37.3	57.4	17	32.8	40.6	42.7	28
	100x	38.5	96.1	90	16.8	33.5	59.7	3.2	23.7	28.6	9.5	50.6	77.1	32.2	105.8	50.1	133.3	44.8	67.6	30.6	26.5	24.3	58.2	28.5
	20x	46.2	97.6	134.5	121.5	15.7	118.3	2.1	74	60.1	26.6	140.1	79.4	77.2	107.8	157.2	135.6	120.6	65.1	45.1	60.4	58.4	98.8	31
	10x	96.5	98.3	72.7	91.8	253.8	115.9	4.7	95.3	72.1	43.3	70.5	124.5	105.7	122.6	100.4	137.1	114.8	64.3	106.2	59.2	78.6	89.3	33.7
	2x	87.2	99.6	83.3	103.8	40	129.7	17.9	132.2	79.5	64.5	112.1	86.7	115.6	121	120.3	128.4	108	64.2	93.4	59.5	91.9	92.2	33.2
	1x	156	109.1	77.7	131.8	52.6	78.7	33.4	100.9	85	75.4	110	85.4	114.2	112.8	119.2	105.1	119.7	68.4	133.1	94.3	118.5	114	36.7
P4VP	A	118.5	98.6	89.4	115.6	45.3	23.1	24.2	110.6	97.5	69.3	84.1	105.9	45.6	122.1	4.6	4.9	138.7	67.6	91.8	32.5	83.8	72.1	3.8
	B	71.6	119.7	94.6	132.2	33.1	40.3	33.2	121.4	115	54.5	103	75.3	96.2	119.9	6.8	36.1	152.2	65.6	87	54.1	108.2	116.4	24.2
	C	52.4	115.1	162.4	103.8	41	27.2	34.8	128.8	111.1	57.3	96.2	83.1	65.1	124.3	5.9	12.1	112.6	61.5	112.1	65.4	111.6	99.5	6.3
	D	66.6	106.3	119.4	143.5	23.4	29.1	26.9	92.1	125.2	50	110.3	86.5	57.6	116.4	8.6	19.1	132.1	62.2	105.2	54.4	87.6	92.2	3.8
Me-QP4VP	A	106.8	121.7	111.5	114.8	48.2	83.1	27.9	122.6	97.2	53.3	88.1	101.4	106.1	135.3	190.8	11.4	121.8	48.3	65.5	13.5	101.4	37.5	3.9
	B	90.4	108.8	80.7	102.5	79.1	154.4	31.5	126.7	98	71.7	117.9	97.7	97.2	126.1	216.4	30.7	114.5	49.9	50.8	13.1	79.9	42.3	3.4
	C	120.7	106.9	116	138.7	46.1	53.8	23.2	135.6	112.2	59.2	117	108.2	100.7	122.3	184.6	44.1	158.4	55.4	63.7	14.4	85.6	40.8	3.4
	D	110.2	110.6	136.1	118.4	86	99	38.6	110	108.1	95.8	112.8	130.9	104.9	131.2	120.2	49.1	148.2	50.4	87.4	11.3	110.2	33.7	2.7
Et-QP4VP	A	111.4	132.6	109.6	144.1	33.1	64.5	26.6	126.8	98.1	69.8	82.3	118.8	122.4	120.3	109.3	90.3	158.1	45.8	68.7	4.2	93	36.6	2.6
	B	131.4	107.5	95	103.5	95.8	90.2	32.2	122.5	128.4	50.4	85.9	121.4	111.1	117.8	141	67.4	137.3	51.5	59.2	8.9	82	43.6	3.1
	C	101.8	114.8	100.5	137.5	91.5	101.5	28.1	108	142.7	59.1	120.6	98.5	101.3	116	138.3	126	150.1	58.7	108.9	4.5	83.4	43	2.8
	D	97.8	115.3	88	129.9	83.1	105	25.6	94.9	103	48	114.8	121.7	96.4	127.7	57.2	7838.4	120	46.9	72.1	8.9	89.8	42.6	2.6
Pr-QP4VP	A	84.9	99.6	100.5	138.3	34.7	100.2	24.4	110.6	100.3	44.7	75.9	90.2	101.8	125.7	69.3	31.4	128.5	23.3	37.8	5.9	78.5	25.7	4.6
	B	142.4	106.1	102.9	126.2	54.4	119.6	49	120.7	122.1	50.4	118.9	93.2	104	130.9	81.8	156.6	105.9	38.4	41	4.3	59.6	35.4	4.4
	C	87.1	128.1	94.3	119.9	29	95.7	35.4	92.5	113.7	43.1	118.1	114.5	120	124.2	84.3	32.3	100.2	30.7	52.9	7	76.4	27.7	4.1
	D	104.3	97.2	117.1	99	39.4	61.8	35.2	123.4	130.4	92.4	111.3	129.8	107.7	129.8	53.2	74.5	116	34.8	56.7	9.4	55.9	34.1	6.4
Feed	1000x	1.9	18.4	18.4	2.9	10.4	16.7	4.8	2.3	3.2	4.1	5.5	11.7	6	41.2	7.2	23.2	9.5	17.3	6.4	10.3	14.4	24.9	7.9
	200x	8.6	91.2	31.8	7.7	52.6	45.9	6.2	11.6	19.4	3.3	23.3	29.7	14.7	140.9	25.1	125.7	43.5	59.8	29.7	27.1	31.3	44.5	23.2
	100x	29.5	97.3	55.8	13.9	76.7	38.6	2.1	13.4	43.7	8.8	41.6	59.7	20	104.9	35.9	116.5	14.1	58.7	45.4	35.9	67.3	76	26.5
	20x	31	129.2	131	71.7	100.1	99	3.2	69	225.3	42.3	82.6	123.4	96.6	106.9	149.3	249.9	138	58.9	64	51.9	84.1	92.4	27
	10x	109.5	107.6	168.4	248.3	103.3	89.1	4.2	122.6	445.3	50.5	127.4	123.9	105.3	108.1	183.2	102.3	100.2	70.6	79.6	61.9	82.6	107.4	27.9
	2x	97.4	110.4	118.3	124.4	102.9	107.7	20.8	109.4	107.3	83.4	118	139.4	117.6	109.8	122.1	99.8	100.7	70.7	96.3	65.2	124.3	102.3	46.3
	1x	126.9	135.3	121.8	152.8	63.1	124.9	27	138.9	146.3	68.4	112.3	105.4	108.3	122.8	124.6	122.4	156.5	63.2	127.1	90.3	122.1	97.6	59.4

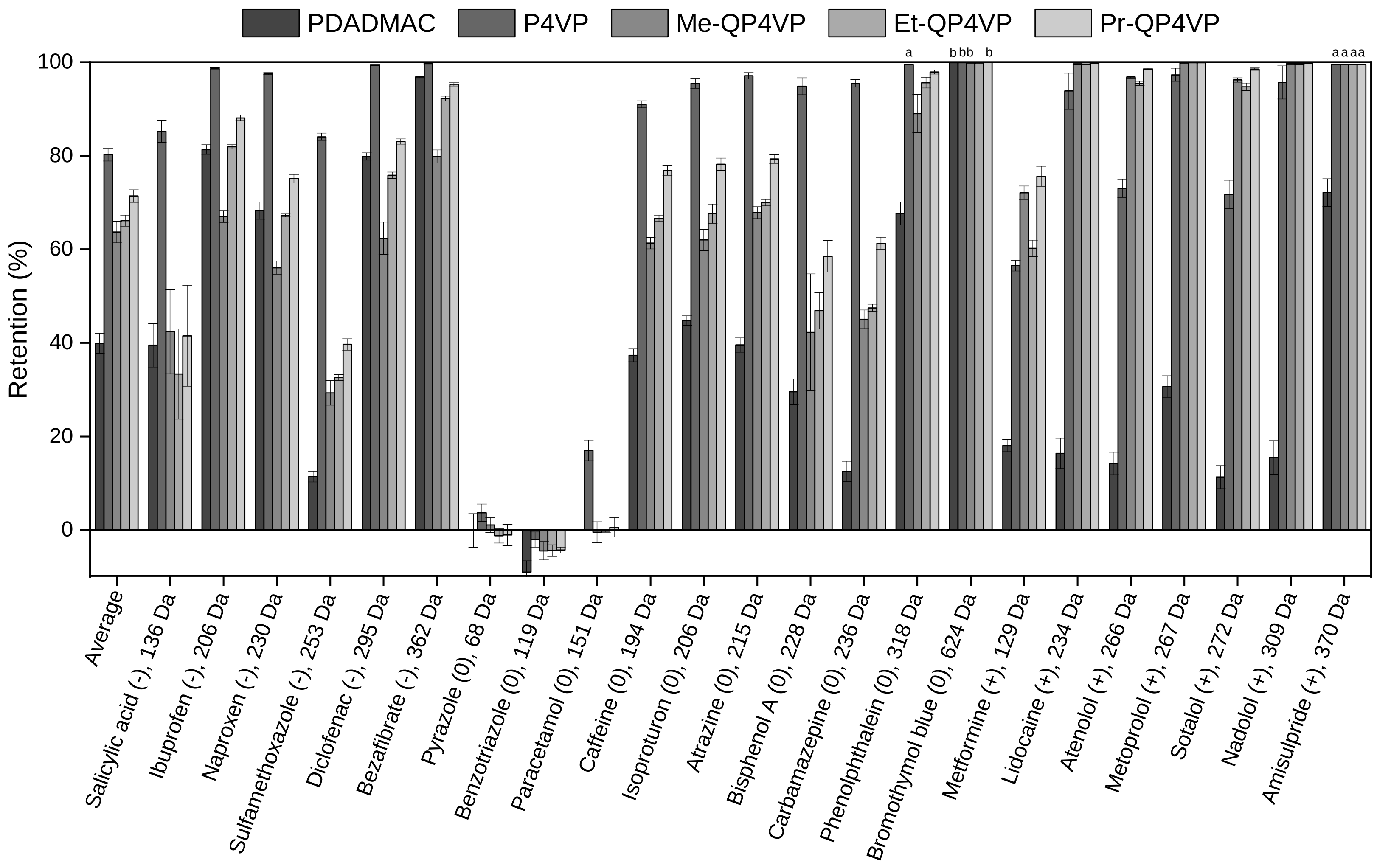

Figure S8: Organic micropollutant (OMP) retention of $[PDADMAC/PSS]_8$, $[P4VP/PSS]_{16.5}$, $[Me\text{-}QP4VP/PSS]_{9.5}$, $[Et\text{-}QP4VP/PSS]_{9.5}$ and $[Pr\text{-}QP4VP/PSS]_{9.5}$ membranes. In some cases, the OMP retention was above the quantification limit. This is indicated with a marker (quantification limits: a = 99.5% and b = 99.9%). The displayed retention is the quantification limit, actual OMP retention may be higher. Error bars represent the 95% confidence interval (n = 4).

8. References

[1] J. de Grooth, R. Oborný, J. Potreck, K. Nijmeijer, W.M. de Vos, The role of ionic strength and odd–even effects on the properties of polyelectrolyte multilayer nanofiltration membranes, Journal of Membrane Science 475 (2015) 311-319. https://doi.org/10.1016/j.memsci.2014.10.044.

[2] D. Gregurec, M. Olszyna, N. Politakos, L. Yate, L. Dahne, S.E. Moya, Stability of polyelectrolyte multilayers in oxidizing media: a critical issue for the development of multilayer based membranes for nanofiltration, Colloid and Polymer Science 293(2) (2015) 381-388. https://doi.org/10.1007/s00396-014-3423-5.
